# Heterologous rhamnolipid biosynthesis by *P. putida* KT2440—ACP dependency and the role of fatty acid metabolism

**DOI:** 10.1128/aem.00930-25

**Published:** 2025-10-21

**Authors:** Melanie Filbig, Philipp Otzen, Matti Froning, Hannah Braß, Alessandra Mauri, George Guo-Qiang Chen, Heiko Hayen, Till Tiso, Lars M. Blank

**Affiliations:** 1 iAMB - Institute of Applied Microbiology, ABBt - Aachen Biology and Biotechnology, RWTH Aachen University 9165, Aachen, Germany; 2 Institute of Inorganic and Analytical Chemistry, University of Münster 9185, Münster, Germany; 3 Lab of Microbiology, School of Life Sciences, Tsinghua University 12442, Beijing, China; 4 Systems Biotechnology, Technical Faculty, Bielefeld University 9167, Bielefeld, Germany; Kyoto University, Kyoto, Japan

**Keywords:** *Pseudomonas putida*, rhamnolipid, biosurfactants, FASII, β-oxidation, ^13^C-labeling, GC-MS, LC-MS, metabolic engineering

## Abstract

Rhamnolipids are glycolipids and, as such, biosurfactants with high surface activity, good biodegradability, low toxicity, and thus manifold applications. Although rhamnolipids have been extensively researched and are already produced industrially, the biosynthesis pathway has not yet been resolved beyond doubt. While the origin of the hydrophilic moiety is clear and all responsible genes and enzymes have been identified, the origin of the hydrophobic moiety, 3-(3-hydroxyalkanoyloxy) alkanoic acid, remains to be identified. Here, the involvement of the two central metabolic pathways, that is, fatty acid *de novo* synthesis (FAS) and β-oxidation in the biosynthesis of rhamnolipids from different carbon sources was investigated in the heterologous production host *Pseudomonas putida* KT2440. Besides using mutants deficient in β-oxidation for this purpose, the absolute configuration, that is, the spatial arrangement of the substituents at the stereocenter of the produced rhamnolipid molecules, was investigated for *P. putida* KT2440, as well as for the native rhamnolipid producer *Pseudomonas aeruginosa* PAO1. Furthermore, ^13^C labeling experiments were performed to gain further insights into the metabolic network. The combined results suggested that the acyltransferase RhlA shows a strong preference for ACP-activated substrates, and that fatty acids are broken down to acetyl-CoA before the C_2_-fragments can be incorporated into rhamnolipids via FAS.

**IMPORTANCE:**

This study contributes to resolving the incomplete understanding of rhamnolipid biosynthesis, particularly the origin of its hydrophobic moiety. Although rhamnolipids have been extensively studied and are already produced industrially, gaps in knowledge regarding their metabolic pathway limit further optimization for large-scale, sustainable production. By investigating the role of fatty acid *de novo* synthesis and β-oxidation in rhamnolipid biosynthesis, this study provides crucial insights that could enable metabolic engineering strategies to enhance production efficiency, especially in the heterologous host *Pseudomonas putida* KT2440, as this strain is considered a safer and more suitable alternative to *Pseudomonas aeruginosa*. These findings collectively pave the way for improved biotechnological production of rhamnolipids, facilitating their broader industrial application as environmentally friendly biosurfactants.

## INTRODUCTION

Rhamnolipids are glycolipids composed of one (mono-RL) or two (di-RL) molecules of L-rhamnose as hydrophilic and one molecule of 3-(3-hydroxyalkanoyloxy) alkanoic acids (HAA) as hydrophobic moiety. This amphiphilic structure qualifies rhamnolipids as molecules exhibiting surface activity. Since rhamnolipids are produced by microorganisms, they are classified as biosurfactants. Due to their special characteristics and physicochemical properties (1, 2), those molecules have several applications. Typical applications as biosurfactants involve detergents, as well as cleaning agents, but also products of the cosmetics and food industry (3). Besides, rhamnolipids may also find application in the pharmaceutical industry, as many medical applications have been proposed (4). Rhamnolipids are mainly produced by representatives of the genus *Pseudomonas* and *Burkholderia* (5). Rhamnolipids are produced as a mixture of congeners, that is, HAA molecules with different chain lengths (6). A typical mixture produced with *Pseudomonas aeruginosa* PAO1 contains, besides HAAs and di-rhamnolipids, mono-rhamnolipids containing one C_8_ and one C_10_ chain (Rha-C_8_-C_10_), two C_10_ chains (Rha-C_10_-C_10_), one C_10_ and one C_12_ chain, containing an unsaturation (Rha-C_10_-C_12:1_) and one C_10_ and one C_12_ chain (Rha-C_10_-C_12_) (7). The molecules thus consist of 24, 26, or 28 C-atoms, respectively, of which six are found in the rhamnose molecule. The molecular weights of the corresponding molecules are listed in Table 1.

**TABLE 1:** HAA and mono-rhamnolipid species produced by *P. aeruginosa* PAO1 and *P. putida* KT2440 SK4 with their C-atoms and molecular weights

	HAA				mono-RL			
Congener	C_8_-C_10_	C_10_-C_10_	C_10_-C_12:1_	C_10_-C_12_	Rha-C_8_-C_10_	Rha-C_10_-C_10_	Rha-C_10_-C_12:1_	Rha-C_10_-C_12_
C-atoms in hydrophob. part	18	20	22	22	18	20	22	22
C-atoms in rhamnose	n.a.^*a*^	n.a	n.a	n.a	6	6	6	6
Molecular weight	330	358	384	386	476	504	530	532

^*a*^ n.a, not applicable.

Although rhamnolipids were mentioned for the first time in 1962 and more than 2,000 publications focus on rhamnolipids already (https://pubmed.ncbi.nlm.nih.gov/?term=rhamnolipid&timeline=expanded, 24 February 2025), the biosynthesis pathway has not been fully elucidated. Yet, in the best-studied rhamnolipid producer *P. aeruginosa* PAO1, while the last enzymatic steps involved in rhamnolipid biosynthesis are known, the biosynthesis of the hydroxy fatty acid moieties is only partially known.

**Fig 1:**
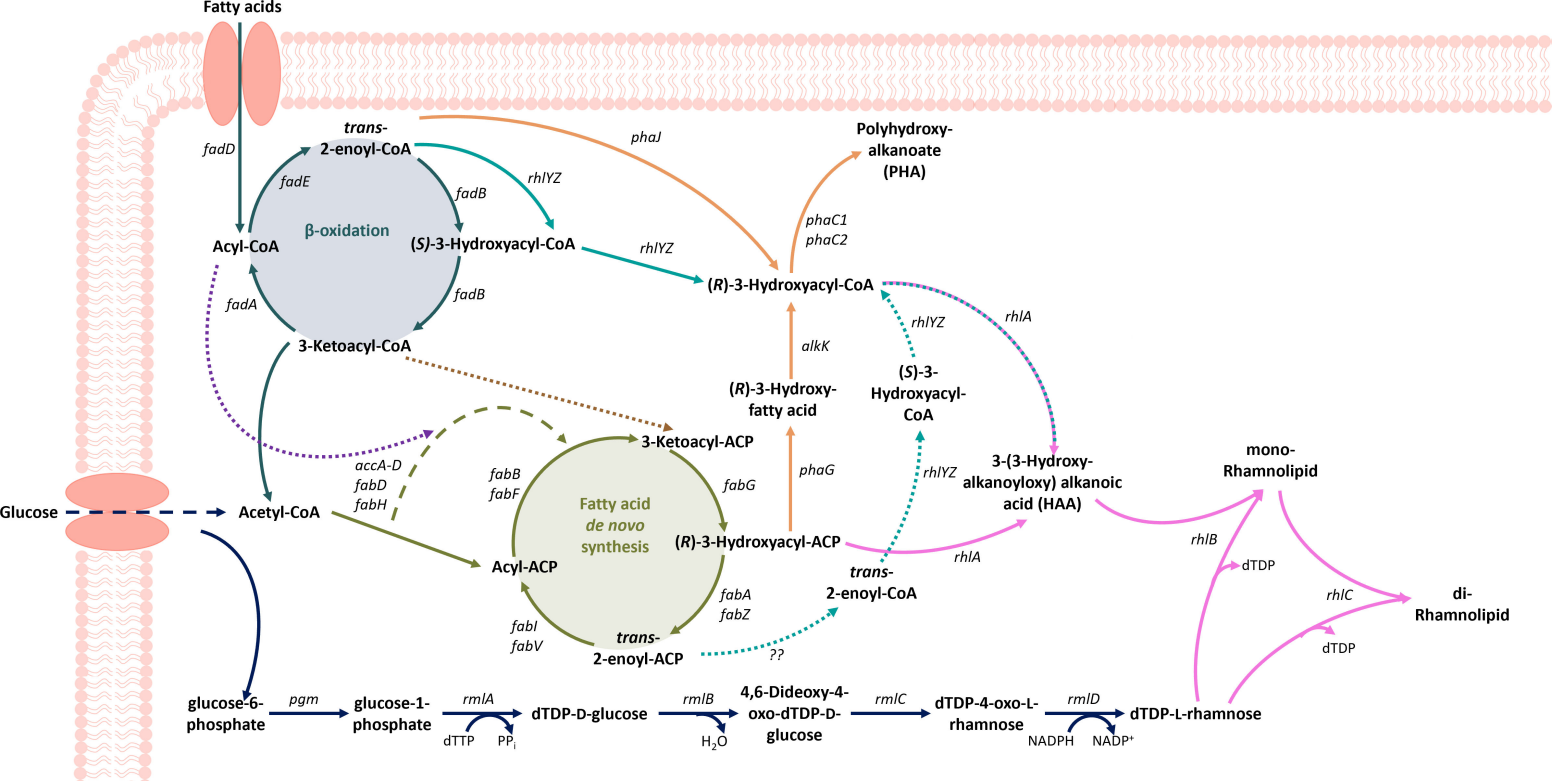
Potential pathways involved in the biosynthesis of rhamnolipids. β-oxidation-related reactions are shown in blue, FAS-related reactions are shown in green, PHA-related reactions are shown in orange, rhamnose biosynthesis is shown in dark blue, and biosynthesis of HAA and rhamnolipid is shown in pink. Dashed lines indicate multiple enzymatic steps, which are not shown in detail. Solid lines represent reactions present in *P. putida* KT2440 SK4, and dotted lines represent connections proposed in the literature. Turquoise lines show a proposed link between β-oxidation and rhamnolipid biosynthesis, provided by RhlYZ, with reactions proposed by Abdel-Mawgoud et al. (15) shown as solid lines, and reactions proposed by Gutiérrez-Gómez et al. (18) shown as dotted lines.

RhlA, an acyltransferase, catalyzes the dimerization of two activated hydroxy fatty acids, resulting in HAA (8). These HAAs are connected to one molecule of L-rhamnose by the activity of the rhamnosyltransferase RhlB using dTDP-L-rhamnose as substrate to form a mono-rhamnolipid (9). RhlC, a second rhamnosyltransferase, then catalyzes the attachment of a second rhamnose using another dTDP-L-rhamnose, resulting in a di-rhamnolipid (10) (Fig. 1). Rhamnolipid biosynthesis thus relies on the synthesis of two precursor molecules derived from central carbon metabolism: dTDP-L-rhamnose and dimerized (*R*)-3-hydroxy fatty acids. The origin of the L-rhamnose moiety is known, and all involved genes and enzymes are identified (11). First, α-D-glucose-6-phosphate is converted to D-glucose-1-phosphate (G1P) by phosphoglucomutase. From G1P, four consecutive enzymatic reactions catalyzed by RmlA, RmlB, RmlC, and RmlD yield dTDP-L-rhamnose, which can then be incorporated into rhamnolipids by RhlB and RhlC (Fig. 1). The biosynthesis route of the fatty acid residue has been the subject of interest in many studies (12–16), but the exact fatty acid precursor has not yet been identified beyond doubt (17). Generally, the fatty acid precursors can originate either from β-oxidation in the form of (*S*)-3-hydroxyacyl-CoA or from fatty acid *de novo* synthesis (FAS) in the form of (*R*)-3-hydroxyacyl-ACP. Molecules deriving from either of the two central metabolic cycles differ in their stereochemical configuration, as well as in their activation. Besides the two metabolic routes delivering potential lipid precursors, the specificity of RhlA toward one or both of them is a key factor.

While M. M. Burger et al. (12) identified CoA-activated hydroxy fatty acids as substrates of RhlA in studies with crude RhlA extracts, K. Zhu and C. O. Rock (14) used purified RhlA and identified ACP-activated hydroxy fatty acids derived from FAS as the direct HAA precursor. In the latter study, β-hydroxydecanoyl-CoA was not found to be a substrate when using RhlA *in vitro* for HAA formation. A combination of both routes, fatty acid degradation and FAS, was also proposed in several studies. A possible link between β-oxidation and FAS was proposed by K. Hori et al. (16): the authors identified β-oxidation to be involved in the synthesis of the lipid moieties of rhamnolipids using odd-length fatty acids, but since RhlA was known to accept only ACP-activated hydroxy fatty acids as substrates, also suggested a link from 3-ketoacyl-CoA from β-oxidation to 3-ketoacyl-ACP in FAS (Fig. 1). A more recent study came to similar conclusions. In experiments with deuterium-labeled octadecanoic acid and glucose, a combination of initial degradation of fatty acids by β-oxidation and subsequent elongation by FAS, as well as a possible shunt between the two routes, was proposed (13).

β-oxidation was found to be the main supplier for HAA-precursors in *P. aeruginosa* PA14 in experiments with deuterium-labeled dodecanoic acid. The results were also confirmed under conditions inhibiting β-oxidation, which resulted in a drastic reduction of the rhamnolipid titer, even when using non-fatty acid substrates, that is, glycerol, implying one of the intermediates of β-oxidation is converted to (*R*)-3-hydroxy fatty acids, which contradicts previous findings (15). The authors thus suggest the existence of a connection between β-oxidation and the rhamnolipid biosynthesis pathway, which is probably executed by RhlYZ, an (*R*)-specific enoyl-CoA hydratases/isomerase (ECH/I) complex converting 2-decenoyl-CoA into (*R*)−3-hydroxydecanoyl-CoA, and which delivers the majority of the hydrophobic rhamnolipid moiety (18) (Fig. 1).

Nevertheless, although the involvement of RhlYZ in rhamnolipid biosynthesis in *P. aeruginosa* was demonstrated, the exact function of the enzymes remains unclear.

Another enzyme discussed to play an important role in rhamnolipid biosynthesis in *P. aeruginosa* is RhlG, a β-ketoacyl reductase, suggested to detract fatty acid precursors from central carbon metabolism towards rhamnolipid biosynthesis (19). However, these findings were contradicted by successive studies. D. J. Miller et al. (20) proposed a role of RhlG in rhamnolipid biosynthesis but not to distract lipid precursors from fatty acid synthesis, since the activity of RhlG with ketoacyl-ACP is too low to compete with FabG. In contrast, K. Zhu and C. O. Rock (14) were not able to assign RhlG any role in rhamnolipid biosynthesis upstream of RhlA since a *rhlG*-deficient mutant of *P. aeruginosa* PA14 still produced wild-type levels of rhamnolipids.

Taken together, those recent results suggest β-oxidation as the main supplier of RL precursors, directed toward rhamnolipid biosynthesis by an additional metabolic link in *P. aeruginosa*, while the specificity of RhlA could not be assigned without doubt. In other native rhamnolipid producers, however, this was shown not to be the case. V. U. Irorere et al. (21) used deuterium labeling to identify FAS as being responsible for delivering the HAA precursor molecules from fatty acids, as well as glycerol, a non-fatty acid-related product, in *Burkholderia thailandensis* E264.

**Fig 2:**
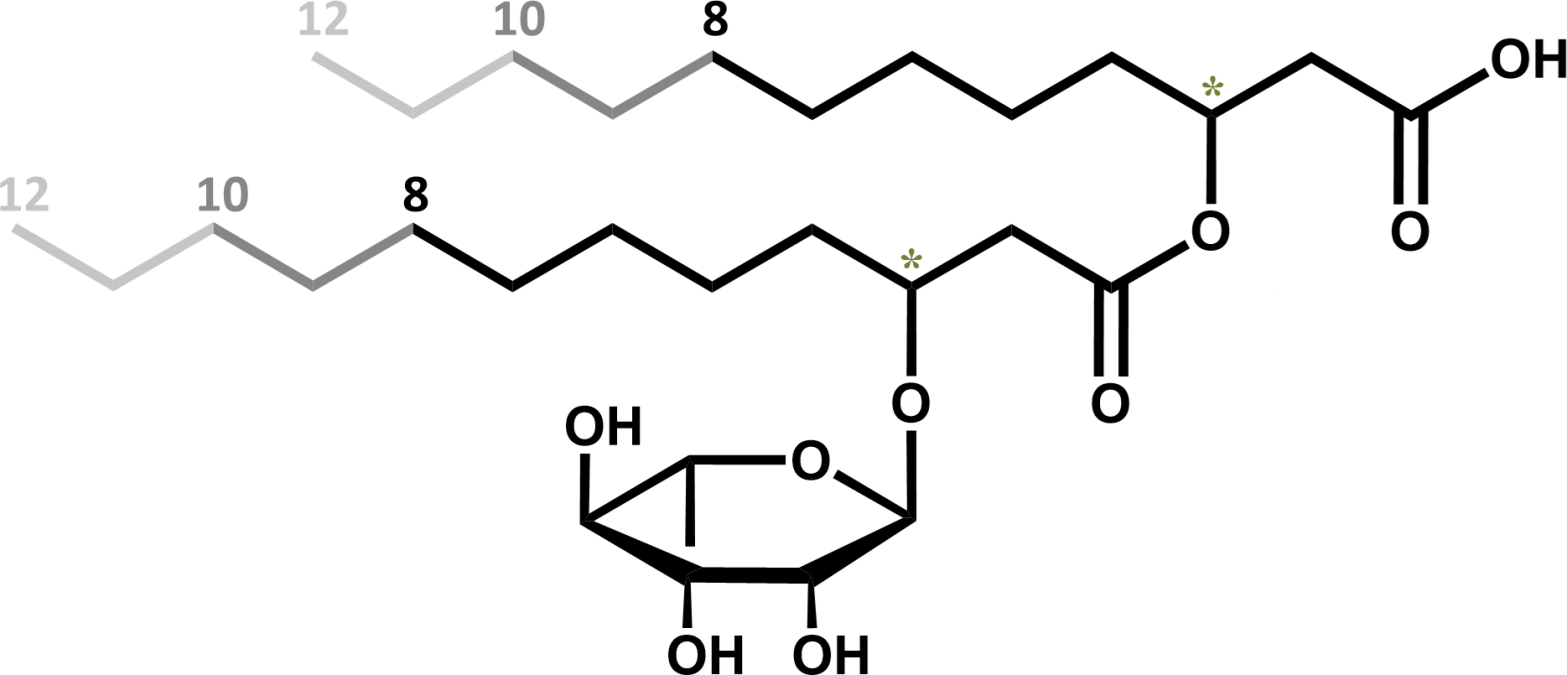
Molecular structure of a mono-rhamnolipid with hydroxy fatty acids containing 8– 12 C-atoms. The asterisks (*) represent stereocenters.

Since activated hydroxy fatty acids deriving from FAS or β-oxidation have a typical configuration, depending on their origin, this information might be indicative of their origin. While hydroxy fatty acids derived from FAS are expected to be in (*R*)-configuration, molecules derived from β-oxidation are expected to be in (*S*)-configuration (12, 14). Thus, another approach used to identify the biosynthesis pathway of rhamnolipids is analyzing the stereochemistry of rhamnolipids, which contain a chiral center in the C_3_-position of each of the 3-hydroxyfatty acid chains (Fig. 2).

Rhamnolipids produced by *P. aeruginosa* have been found to exhibit the same stereochemistry in the 3-hydroxyfatty acids as in fatty acid biosynthesis and as found in the storage polymer polyhydroxyalkanoates (PHAs), which is the (*R*)-configuration and matches the one from FAS (14, 17, 22). Since PHAs, as well as HAA molecules, consist of activated (*R*)-3-hydroxy fatty acids and thus are structurally related, the synthesis of PHAs might also give a hint at HAA synthesis. When PHAs are produced from fatty acid-related substrates, an (*R*)-specific enoyl-CoA hydratase catalyzes the hydration of trans-2-enoyl-CoA derived from β-oxidation to (*R*)-3-hydroxyacyl-CoA (23), indicating that first, analysis of the stereochemistry can only give hints at the exact biosynthesis pathway and second, β-oxidation and FAS are interconnected.

*P. putida* KT2440 has frequently been used as a non-pathogenic heterologous rhamnolipid producer (24–36), but the biosynthesis pathway of the lipid moiety is also not known in this strain. Overall, the metabolism of *P. putida* KT2440 is similar to the metabolism of the native producer *P. aeruginosa* PAO1, which becomes evident in the fact that it is sufficient to heterologously express the genes encoding the acyltransferase RhlA and rhamnosyltransferase RhlB to enable rhamnolipid biosynthesis. A mutant of *P. putida* KT2440 deficient in β-oxidation, *P. putida* KTQQ20 (37) was shown to still be able to produce PHAs from fatty acid substrates *via* the (*R*)-specific enoyl-CoA hydratase, catalyzing the hydration of trans-enoyl-CoA toward (*R*)-3-hydroxyacyl-CoA, the precursor of the PHA polymerase PhaC. Since PHA and HAA probably share a common pool of precursor molecules, it might be possible that *P. putida* KT2440 uses the described enzyme also for the biosynthesis of the rhamnolipid lipid moiety.

To fully exploit the potential of *P. putida* KT2440 as a heterologous rhamnolipid producer and optimize the strain’s metabolism, the biosynthesis pathway must be completely identified.

In this study, we investigated the biosynthesis pathway of the rhamnolipid-lipid moiety of the heterologous rhamnolipid producer *P. putida* KT2440. The stereochemical configuration of rhamnolipids produced by *P. putida* KT2440 was analyzed using gas chromatography-mass spectrometry (GC-MS) after hydrolysis and derivatization of the liberated 3-hydroxy fatty acids with Mosher’s reagent. Furthermore, genetically engineered strains, as well as ^13^C-labeled substrates, were utilized to determine ^13^C incorporation into intact rhamnolipid molecules monitored by liquid chromatography-tandem mass spectrometry (LC-MS/MS). The results are discussed in the context of the literature, to shed light in the dark.

## MATERIALS AND METHODS

### Bacterial strains and cultivation conditions

Strains and plasmids used in this study are described in Table 2. Bacterial strains were routinely cultivated in lysogeny broth (10 g L^−1^ peptone, 5 g L^−1^ yeast extract, and 10 g L^−1^ NaCl). 1.5% (wt/vol) agar was added to the medium to prepare solid medium. While *P. putida* strains were grown at 30°C, strains of *E. coli* were cultivated at 37°C. 50 µg mL^−1^ kanamycin, 30 µg mL^−1^ gentamycin, or 20 µg mL^−1^ tetracycline was added to the medium for selection purposes or to avoid plasmid loss. Cetrimide agar (Sigma-Aldrich, St. Louis, MO, USA) was used to select for cells of *Pseudomonas* after mating procedures. A mineral salt medium with a final composition (per L) of 1.55 g K_2_HPO_4_, 0.85 g NaH_2_PO_4_·2H_2_O, 2.0 g (NH_4_)_2_SO_4_, 0.1 g MgCl_2_·6H_2_O, 10 mg EDTA, 2 mg ZnSO_4_·7H_2_O, 1 mg CaCl_2_·2H_2_O, 5 mg FeSO_4_·7H_2_O, 0.2 mg Na_2_MoO_4_·2H_2_O, 0.2 mg CuSO_4_·5H_2_O, 0.4 mg CoCl_2_·6H_2_O, and 1 mg MnCI_2_·2H_2_O (38) was used for the production of rhamnolipids. For the analysis of labeled amino acids, *P. putida* KT2440 pPS05 was cultivated in M9 minimal medium with a final composition (per L) of 8.5 g Na_2_HPO_4_·2H_2_O, 3 g KH_2_PO_4_, 0.5 g NaCl, 1 g NH_4_Cl, 2 mM MgSO_4_, 4.87 mg FeSO_4_·7H_2_O, 4.12 mg CaCl_2_·2H_2_O, 1.5 mg MnCl_2_·4H_2_O, 1.87 mg ZnSO_4_·7H_2_O, 0.3 mg H_3_BO_3_, 0.25 mg Na_2_MoO_4_·2H_2_O, 0.15 mg CuCl_2_·2H_2_O, and 0.84 mg Na_2_EDTA·2H_2_O (39). Glucose was added in a concentration of 0.33 C-mol for pre-cultures. For main cultures, glucose or uniformly isotope-labeled U-^13^C_6_ glucose (Cambridge Isotope Laboratories, Inc., Tewksbury, MA, USA) was used in concentrations of 0.17 C-mol or 0.33 C-mol. Sodium octanoate, sodium decanoate, and sodium dodecanoate were used in concentrations equivalent to 0.17 C-mol or 0.33 C-mol. Sampling experiments were performed in shake flasks with 10% filling volume. A Growth Profiler 960 (Enzyscreen B.V., Heemstede, The Netherlands) was used for online growth analysis with endpoint sampling in 24-deep well plates with transparent bottom with 1.5 mL filling volume at 225 rpm with a throw of 50 mm. The corresponding Growth Profiler software determined the density of the culture by image analysis. Online monitoring of oxygen and carbon dioxide transfer rates was performed in shake flasks using the Kuhner TOM system (Adolf Kühner AG, Birsfelden, Switzerland). A total carbon content of 0.17 C-mol L^−1^ was applied in a filling volume of 9%, and cultivation was performed at 300 rpm.

**TABLE 2:** Bacterial strains used in this study

Strains and plasmids	Characteristics	References
*E. coli*		
PIR2	F, Δ*lac169*, *rpoS*(*Am*), *robA1*, *creC510*, *hsdR514*, *endA*, *recA1*, *uidA* (Δ*MluI*)::pir; host for *oriV*(R6K) vectors	ThermoFisher Scientific
HB101 pRK2013	Sm^R^*, hsdR-M*^+^, *proA2*, *leuB6*, *thi-1*, *recA*; harboring plasmid pRK2013	Ditta et al. (40)
DH5α pSW-2	*supE44*, ∆*lacU169* (*Φ80lacZ*ΔM15), *hsdR17* (r_K_^−^ m_K_^+^), *recA1*, *endA1*, *thi-1*, *gyrA96*, *relA1*; harboring plasmid pSW-2 encoding I-SceI nuclease, tool for genomic deletion	Martínez-García and V. de Lorenzo (41)
DH5αλpir pTnS-1	λ*pir* lysogen of DH5α; harboring plasmid pTnS**-**1	H. Choi et al. (42)
DH5αλpir pSK02	DH5αλ*pir* harboring Tn7 delivery vector pSK02 for chromosomal integration, containing *rhlAB* genes from *P. aeruginosa* PA01	Bator et al. (34)
PIR2 pBG-ffg-*rhlAB-fadB*	PIR 2 harboring Tn7 delivery vector pSK02 for chromosomal integration; containing *rhlAB* genes from *P. aeruginosa* PA01 and *fadB* (PP_2136) from *P. putida* KT2440	This study
PIR2 pEMG-PP_3754-55	PIR2 harboring plasmid pEMG-PP_3754-55	This study
*P. putida*		
KT2440	Wild type	Bagdasarian et al. (43)
KT2440 ΔPP_3754-55	ΔPP_3754-55	This study
KTQQ20	KT2442 Δ*fadB*, Δ*fadA*, Δ*fadB2x*, Δ*fadAx*, Δ*PP2047*, Δ*PP2048*, Δ*phaG*	Liu et al. (37)
KTQQ20 ΔPP_3754-55	KTQQ20 ΔPP_3754-55	This study
KT2440-RL	KT2440 with *att*Tn7::P_ffg_-*rhlAB*	Bator et al. (34)
KT2440 ΔPP_3754-55-RL	KT2440 ΔPP_3754-55 RL with *att*Tn7::P_ffg_-*rhlAB*	This study
KTQQ20 RL	KTQQ20 with *att*Tn7::P_ffg_-*rhlAB*	This study
KTQQ20 ΔPP_3754-55-RL	KTQQ20 ΔPP_3754-55 with *att*Tn7::P_ffg_-*rhlAB*	This study
KTQQ20 ΔPP_3754-55-RL-*fadB*	KTQQ20 ΔPP_3754-55 with *att*Tn7::P_ffg_-*rhlAB-fadB*	This study
KT2440 pPS05	KT2440 harboring plasmid pPS05 (pSynPro16, *rhlAB*, Tet^R^)	Tiso et al. (28)

### Plasmid cloning and strain engineering

The NEBuilder Assembly online tool was used to plan plasmid construction, which was performed using the NEBuilder HiFi DNA Assembly Kit (New England Biolabs, Ipswich, MA, USA). DNA fragments were amplified using Q5 High-Fidelity DNA Polymerase (New England Biolabs, Ipswich, MA, USA) according to the manufacturer’s instructions. Primers were ordered as custom DNA oligonucleotides (Eurofins Genomics, Ebersberg, Germany). 5′-agggataacagggtaatctgGTTCGACTGCACGCTGATC-3′ and 5′- cgcttgctccCTCCGATTGCGAAGGCGTG-3′ were used to amplify TS1 of PP_3754-55, 5′-gcaatcggagGGAGCAAGCGCCATGCCC-3′ and 5′- atccccgggtaccgagctcgTCAGCTCCAAGCCCTGCTC-3′ were used to amplify TS2 of PP_3754-55. pSK02 was linearized using 5′-GAATTCGAGCTCGGTACCC-3′ and 5′- tccatcttctccttgttgcggcccgacgtcgcatgctcctctagactcgaggaattcgcttcggtctTCAGGACGCAGCCTTCAG-3′ and assembled with *fadB*, amplified with 5′- agaccgaagcgaattcctcgagtctagaggagcatgcgacgtcgggccgcaacaaggagaagatggaATGATTTACGAAGGTAAAGCC-3′ and 5′-cgggtaccgagctcgaattcTCAGTTGAAGAAGCGCTGGC-3′. DNA assembly products were transferred into chemically competent *E. coli* PIR cells via heat shock according to the protocol of Hanahan (1983). Colony PCR using One*Taq* 2X Master Mix with Standard Buffer (New England Biolabs, Ipswich, MA, USA) was performed to verify positive clones. Cell material from colonies was lysed with alkaline polyethylene glycol prior to PCR, according to Chomczynski and Rymaszewski (2006). Deletion of PP_3754-55 was controlled using 5′-CCTTTGCCTAGACTCGATCC-3′ and 5′-ACGTCGCTCCTCAAAGTTGG-3′. Integration of genes into the *att*Tn7 site was controlled using 5′-AGTCAGAGTTACGGAATTGTAGG-3′ and 5′-GTCGAGAAAATTGCCGAGCT-3′.

The I-SceI-based system (Martínez-García and de Lorenzo, 2011) was used to create gene deletions. 500 bp regions upstream and downstream of the region to be deleted, referred to as TS1 and TS2 regions, were amplified from genomic DNA of *P. putida* KT2440 (isolated with Monarch Genomic DNA Purification Kit, New England Biolabs, Ipswich, MA, USA) and cloned into the suicide delivery vector pEMG. The conjugation protocol from B. Wynands et al. (44) was used to transfer the resulting pEMG-PP_3754-55 from *E. coli* PIR2 into the respective *Pseudomonas*. Kanamycin-sensitive clones were cured of the pSW-2 plasmid by repeated transfer of cells into fresh LB medium without antibiotics and verified again by colony PCR.

Rhamnolipid producers were generated by chromosomal integration of the *rhlAB* into the *att*Tn7-site using the mini-Tn7 delivery transposon vector of pBG14f_80i_14f_80i_14g (45), as described by I. Bator et al. (34). The mini-Tn7 delivery transposon contained on the plasmid pSK02 was integrated into the genome of mutants of *P. putida* KT2440 *via* transposition. Identification of the rhamnolipid-producing colonies was performed as described by I. Bator et al. (34) before the best-producing colonies were selected from cultivation in LB medium containing 0.33 C-mol glucose.

### Gene expression studies

Samples for RNA sequencing were taken during cultivation in the early exponential phase at an OD_600_ of 1.0–1.5. The corresponding cell count was determined using the BactoBox (SBT Instruments, Copenhagen, Denmark). 1 mL of sample was pelleted by centrifugation at 13,300 rpm for 2 min, and the supernatant was discarded prior to snap-freezing in liquid nitrogen. Samples were stored at −80°C until sent for RNA isolation, rRNA depletion, and RNA sequencing, which was performed by Eurofins Genomics Germany GmbH (Ebersberg, Germany), as well as gene expression analysis.

### Analytical methods

Cell growth was monitored by measurement of the optical density of culture broth at 600 nm (OD_600_) using an Ultrospec 10 cell density meter (Biochrom, Cambridge, UK).

#### Rhamnolipid analysis

For rhamnolipid quantification, samples were taken from liquid cultivation, mixed 1:1 with acetonitrile, and stored at 4°C overnight. Afterward, the mixture was centrifuged at 13,300 rpm for 5 min and filtered with Phenex RC syringe filters (0.2 µm, Phenomenex, Torrance, CA, USA). Reversed-phase chromatography was performed using a Dionex UltiMate 3000 system (Thermo Fisher Scientific, Waltham, MA, USA) consisting of a column compartment, an autosampler, and a dual gradient pump connected to a Corona Veo Charged Aerosol Detector (CAD) (all Thermo Fisher Scientific, Waltham, MA, USA). A NUCLEODUR C18 Gravity column (150 × 3 mm, 3 µm) (Macherey-Nagel, Düren, Germany) was used for chromatographic separation of different congeners. The flow rate in the analytical gradient was set to 0.425 mL min^−1^ at a column oven temperature of 40°C. Gradient elution was performed using the two mobile phases: (A) acetonitrile with 0.2% formic acid (vol/vol) and (B) 0.2% formic acid in ultrapure H_2_O (vol/vol). The gradient was set to 70% A and 30% B at the start. The share of A linearly increased to 80% in the first 8 minutes and remained constant for 1 min, before increasing to 100% until minute 13. After 3 minutes, A was decreased back to 70% in 1.5 min and remained constant until the measurement ended after 25 min. The injection volume was set to 3 µL.

To equalize the altering solvent composition in the CAD and guarantee a constant solvent share of 85%, an inverse gradient was applied, which was calculated by the software in the mode “keep solvent composition” (Chromeleon 7.2.10). The offset was determined experimentally to 778 µL. The share of A in the inverse gradient was 100% in the beginning and decreased to 90% from 1.83 min to 9.83 min. After 10.83 min, the share of A decreased further to 70%, before it increased back to 100% after 19.33 min. 1-monoolein was used to generate a calibration function.

#### Analysis of the stereochemical configuration of fatty acids in rhamnolipids

The stereochemical configuration of rhamnolipids was determined using GC-MS. For sample preparation, acidic hydrolysis of the rhamnolipids was performed first. 0.5 mL of a 2.7 M H_2_SO_4_ and 0.5 mL of CHCl_3_ were added to 500 µL of the culture supernatant. The resulting biphasic system was heated for 120 min at 100°C before the CHCl_3_ phase containing the 3-hydroxy fatty acids was collected and evaporated to dryness under a gentle nitrogen stream. For methylation of the carboxyl group of the hydrolyzed fatty acids of the rhamnolipids and the standards (10 mg of the respective standard), 200 µL of BF_3_ (14% in MeOH) was added to the dried substances. 3 mL H_2_O was added after heating for 60 min at 75°C, and the fatty acid methyl esters were extracted with chloroform (3 × 3 mL). Afterward, the chloroform was removed with a gentle stream of nitrogen, and the transformation of the enantiomers into diastereomers was carried out according to a modified method of R. Jenske and W. Vetter (46). Therefore, 200 µL pyridine and 10 µL Mosher’s reagent were added, and the mixture was allowed to react at room temperature for 120 min. The residues were suspended in 5 mL H_2_O and extracted with methyl *tert*-butyl ether (MTBE, 2 × 5 mL). The combined MTBE layers containing the methylated 3-hydroxy fatty acids as their (*R*)-(-)-α-methoxy-α-(trifluoromethyl)-phenylacetyl chloride (MTPA, Mosher’s reagent) derivatives were collected and evaporated to dryness. For GC-MS analysis, the residues were re-dissolved in 1 mL MTBE. A GCMS-QP-2020 (Shimadzu, Kyoto, Japan) equipped with a Nexis GC-2030 gas chromatograph (Shimadzu, Kyoto, Japan) was used for the analysis of the 3-hydroxy fatty acids. Samples were separated on a 30 m, 0.25 mm i.d., 0.25 µm film thickness DB-5MS column (J&W Scientific, Folsom, CA, USA). Using an AOC-20i Plus autosampler (Shimadzu, Kyoto, Japan) and a PTV inlet (250°C) in split mode (split 1:10), samples (1 µL) were introduced into the system. Helium (5.0) was used as carrier gas with a constant flow rate of 1.20 mL min^−1^. For chromatographic separation, a method from R. Jenske and W. Vetter (47) was adapted, and the column oven was programmed as follows: starting at 60°C and holding this for 1.5 min, the temperature was increased at a rate of 40°C min^−1^ to 180°C (hold time 2 min). Then, the temperature was raised at 2°C min^−1^ to 220°C. After keeping this temperature for 35 min, it was increased to 300°C at a rate of 10°C min^−1^ and held for 5.5 min. Mass spectra were obtained by electron ionization (EI, 70 eV). The temperature of the ion source and interface was set to 250°C and 300°C, respectively. Data were recorded from *m/z* 50–600 with a rate of 10 scans/s. GC-MS solution 4.50 (Shimadzu, Kyoto, Japan) was used for instrument control and data processing.

#### Analysis of ^13^C-labeling in rhamnolipids

^13^C-Labeling experiments were performed with U^13^C_6_-glucose as the sole carbon source or in combination with fatty acids. Samples for LC-MS measurement were taken after depletion of the carbon source(s). Therefore, 6 mL of the culture was mixed with acetonitrile prior to centrifugation and filtration. Analysis of the samples was performed using a Vanquish Flex Duo System (Thermo Fisher Scientific, Waltham, MA, USA) equipped with a Dual Split Sampler FT, a Dual Pump F, and a column compartment H. A NUCLEODUR C18 Gravity column (150 × 2 mm, 3 µm) (Macherey-Nagel GmbH & Co. KG., Düren, Germany) was used for chromatographic separation. The mobile phases consisted of (A) 0.2% formic acid in ultrapure H_2_O (vol/vol) and (B) acetonitrile with 0.2% formic acid (vol/vol). The gradient was applied as follows: 0 min, 40% B; 2 min, 40% B; 7 min, 70% B; 16 min, 80% B; 18 min, 80%; 22 min, 100% with an added cleansing for 3 min at 100% B and an equilibration step for 5 min with 40% B. The gradient was run at a flow rate of 0.3 mL min^−1^ and an oven temperature of 40°C. The injection volume of the samples was set at 5 µL. Mass spectrometric detection was carried out using a hybrid quadrupole-orbitrap mass spectrometer (Q Exactive Plus, Thermo Fisher Scientific, Waltham, MA, USA) in combination with a heated electrospray ionization source (HESI-II). The system was operated in data-dependent mode, resulting in one full scan followed by three MS/MS scans of the most intense ions. The full scan was carried out with a resolution of 70,000 (at *m/z* 200) in the mass range of *m/z* 150–1,000, an automatic gain control (AGC) target of 1e6, and a maximum injection time of 100 ms. MS/MS data were obtained using an HCD cell with a normalized collision energy (NCE) of 30 (based on *m/z* 500), a resolution of 17,500 (at *m/z* 200), an AGC target of 1e5, as well as a maximum injection time of 50 ms. Measurements were executed in negative ionization mode. The quadrupole isolation window was set to ±1.0 Da. Applied settings for HESI-II were as follows: capillary temperature 263°C, heater temperature 200°C, sheath gas flow rate 30 auxiliary units (AU), auxiliary gas flow rate 10 AU, sweep gas flow rate 1 AU, spray voltage –3 kV, and s-lens RF level 53. The system was controlled using the Xcalibur (version 4.1) software with the SII Chromeleon plugin. Peak integration was carried out using the Xcalibur (version 4.1) software. Extracted ion chromatograms (EICs) were obtained with a mass tolerance of 10 ppm (parts per million) and an applied smoothing (boxcar) of 5 data points. Peak integration was accomplished with the Genesis peak integration algorithm. Results are shown as average peak area of all three biological replicates per experimental condition.

#### Amino acid analysis

To determine ^13^C-labeling in amino acids, 0.3 mg CDW was obtained by centrifugation at 13,300 rpm for 5 min, and the pellet was stored at −20°C until preparation for GC-MS measurement. The thawed pellet was washed with double-distilled water and pelleted again prior to resuspension in 150 µL 6 M HCl. Samples were transferred into conical glass vials and incubated at 105°C for 6 h to allow biomass hydrolysis. Samples were subsequently dried at 85°C overnight before 30 µL acetonitrile and 30 µL *N*-methyl-*N*-tert-butyldimethylsilyl-trifluoroacetamide, serving as derivatization reagent, were added, and the reaction was allowed for 1 h at 85°C. Amino acids in the samples were then analyzed by GC-MS (48). GC of amino acids was performed with a Trace GC Ultra equipped with an AS 3000 autosampler on a TraceGOLD TG-5SilMS capillary column (all Thermo Fisher Scientific, Waltham, MA, USA). Chromatographic separation was performed at a constant flow rate of 1 mL min^−1^ helium and a temperature gradient. In the first minute, the temperature was constant at 140°C and afterward increased linearly with 10°C min^−1^ to 310°C, before remaining constant for another minute. 1 µL sample volume was injected in a split/splitless injector at 270°C; the split ratio was set to 1:15. Detection of amino acids was performed with a selected ion monitoring mode. MS analysis was performed on an ISQ single quadrupole mass spectrometer (Thermo Fisher Scientific, Waltham, MA, USA). The temperature of the ion source and the transfer line was set to 280°C. Ionization was performed by electron ionization at 70 eV (48). Raw data from GC-MS were analyzed using Xcalibur and corrected for natural labeling by the use of iMS2Flux.

### Calculation of fractional labeling

The fractional labeling (FL) was used to determine the fraction of carbon atoms in rhamnolipids and amino acids that are ^13^C-labeled and, thus, derived from the labeled substrate, in this case, glucose, in a co-feed of glucose and fatty acids. First, the relative distribution of different isotopomers, that is, molecules differing only in their isotopic composition, was analyzed, resulting in a mass isotopomer distribution (MID). FL was then calculated as the weighted average of the number of labeled carbons in the molecules, normalized by the total number of carbon atoms in the molecule, that is, the total possible labeled atoms:


$$FL=\frac{\sum_{i=1}^{n}i\cdot {MID}_{i}}{n\cdot \sum_{i=0}^{n}{MID}_{i}}$$

where i = number of 13C-atoms in the isotopomer, ${MID}_{i}$ = relative abundance of the i-th isotopomer, and n = total number of carbon atoms in the molecule.

## RESULTS

### Only (*R*)-configuration found in hydroxy fatty acids incorporated in rhamnolipids

*P. putida* KT2440 SK4 and *P. aeruginosa* PAO1 were cultivated on glucose and plant oil and glucose and fatty acids, respectively, to analyze the stereochemistry of the hydroxy fatty acids with a length of 10, 12, or 14 C-atoms and without unsaturation (3-OH-10:0, 3-OH-12:0, and 3-OH-14:0) in rhamnolipids produced from different substrates with GC/EI-MS. 3-OH-8:0 and the monounsaturated 3-OH-12:1 were not analyzed since standards were not accessible. Rhamnolipids purified from the culture broth were hydrolyzed into their free β-hydroxy fatty acids and subsequently transformed into their methylated MTPA derivatives to determine the absolute configuration of the molecules. Analysis of the stereochemistry of hydroxy fatty acids contained in rhamnolipids produced by the native producer *P. aeruginosa* PA01 from glucose and plant oil revealed that, independently of the carbon source, only a fraction was produced in (*S*)-configuration, while the majority was produced in (*R*)-configuration (Table 3). These results are in accordance with previous findings from K. Zhu and C. O. Rock (14).

**TABLE 3:** Stereochemistry of rhamnolipids produced with *P. aeruginosa* PAO1 from glucose and plant oil and with *P. putida* KT2440 SK4 from glucose, decanoate, and tetradecanoate^*a*^

	*P. aeruginosa* PAO1						*P. putida* KT2440 SK4								
Substrate	Glucose			Plant oil			Glucose			Decanoate			Tetradecanoate		
Congener	C_10_	C_12_	C_14_	C_10_	C_12_	C_14_	C_10_	C_12_	C_14_	C_10_	C_12_	C_14_	C_10_	C_12_	C_14_
% (*R*)	99.5	99.6	100	99.8	99.4	100	99.8	99.5	100	100	100	n.d.^*a*^	100	100	n.d.
% (*S*)	0.5	0.4	n.d.	0.2	0.6	n.d.	0.2	0.5	n.d.	n.d.	n.d.	n.d.	n.d.	n.d.	n.d.

^*a*^ n.d., not detected.

While rhamnolipids in (*S*)-configuration have been detected in fractional parts produced by the heterologous producer *P. putida* KT2440 SK4 from glucose (Table 3), only (*R*)-configuration was detected in rhamnolipids produced by this strain from the fatty acids decanoate and tetradecanoate. Thus, independent of the carbon source, rhamnolipids seem to be produced exclusively in (*R*)-configuration in both species.

**Fig 3:**
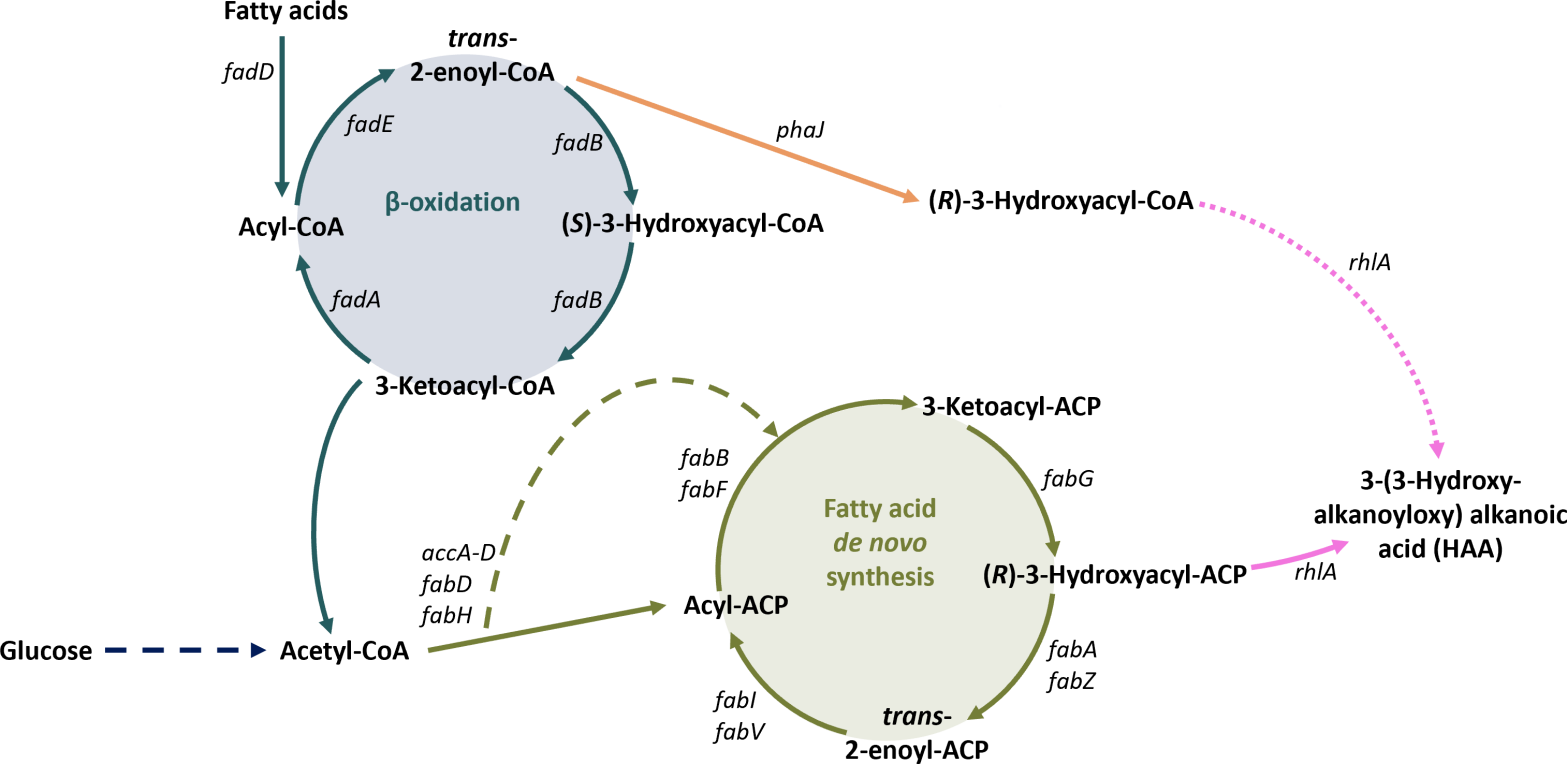
Potential pathways involved in the biosynthesis of 3-(3-hydroxyalkanoyloxy) alkanoic acids from glucose and fatty acids. β-oxidation-related reactions are shown in blue, FAS-related reactions are shown in green, and HAA biosynthesis is shown in pink. Dashed lines indicate multiple enzymatic steps, which are not shown in detail. Solid lines represent reactions present in *P. putida* KT2440 SK4.

These results can give insights into the underlying biosynthesis pathways. When rhamnolipids are synthesized from glucose, hydroxy-fatty acids are generated via FAS from acetyl-CoA, resulting in (*R*)-configuration of ACP-activated hydroxy-fatty acids (Fig. 3). When fatty acids or plant oils are used as substrate for rhamnolipid production, these can either be broken down to acetyl-CoA *via* β-oxidation or directly incorporated into rhamnolipids. In the first case, the resulting acetyl-CoA is used for the synthesis of new fatty acids from C_2_-molecules using FAS, resulting in (*R*)-hydroxyacyl-ACP, which can be used by RhlA for the formation of HAA. Several proposed possibilities exist for the latter case. After dehydrogenation of CoA-activated fatty acids to trans-2-enoyl-CoA, these might be the substrate of PhaJ, an (*R*)-specific enoyl-CoA hydratase, yielding (*R*)-hydroxyacyl-CoA. Since the use of CoA-activated hydroxy-fatty acids as substrate for RhlA has not yet been excluded, these could conceivably be used for HAA synthesis directly or be subject to a trans-activation, that isi.e., the transfer from CoA to acyl-carrier protein as executed by transacylases (Fig. 3).

The presence of rhamnolipids solely containing fatty acids in (*R*)-configuration indicates the degradation to acetyl-CoA, which then enters FAS.

### Rhamnolipids cannot be produced from fatty acids in a β-oxidation-deficient strain

**Fig 4:**
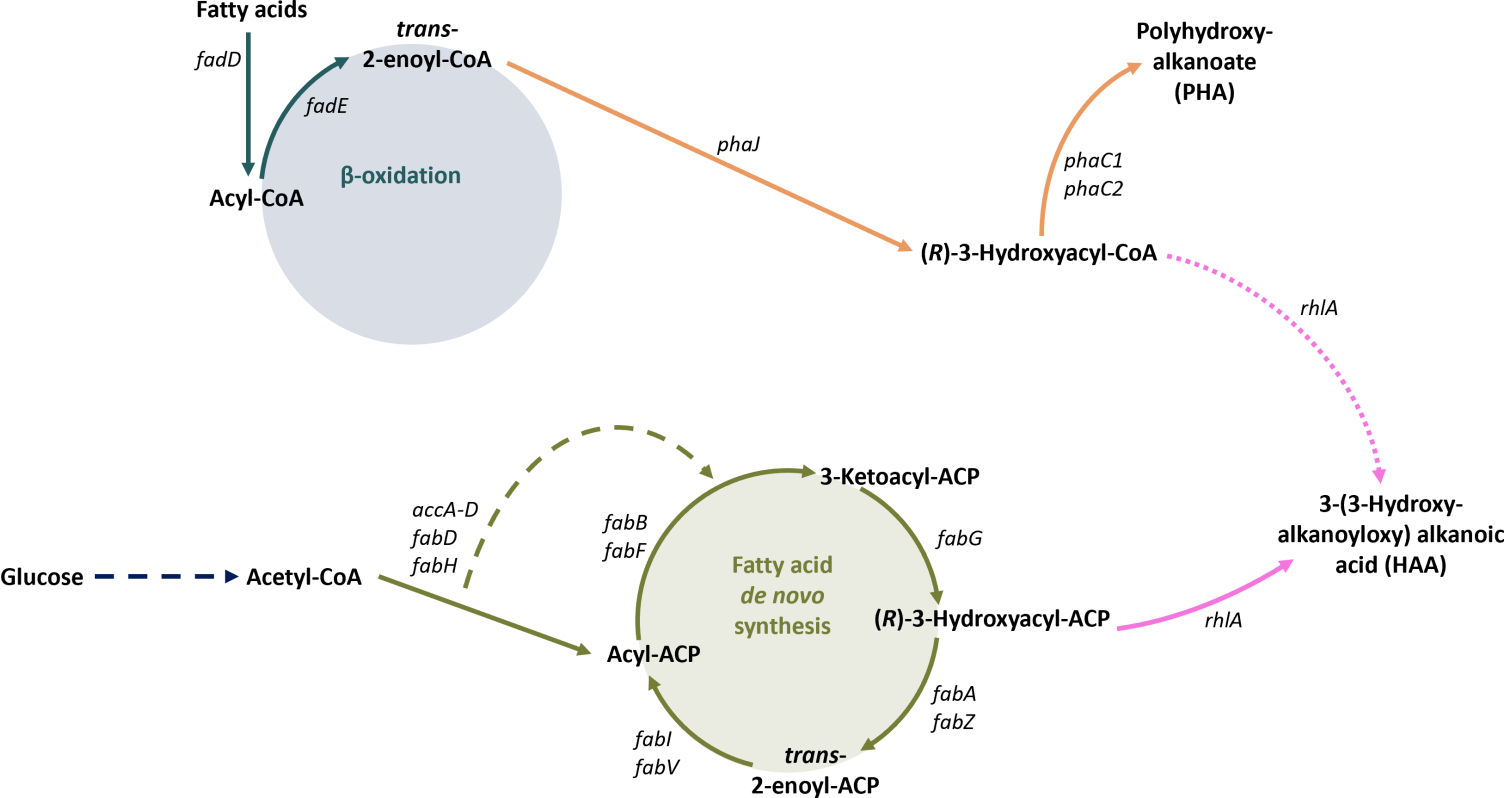
Potential pathways leading to the formation of 3-(3-Hydroxyalkanoyloxy) alkanoic acids in mutant *P. putida* KTQQ20. β-oxidation-related reactions are shown in blue, FAS-related reactions are shown in green, PHA-related reactions are shown in orange, rhamnose biosynthesis is shown in dark blue, and biosynthesis of HAA and rhamnolipid is shown in pink. Dashed lines indicate multiple enzymatic steps, which are not shown in detail. Solid lines represent reactions present in *P. putida* KTQQ20 *att*Tn7::P_ffg_-*rhlAB*.

To further elucidate the involvement of β-oxidation in rhamnolipid production with *P. putida* KT2440 from different carbon sources, strain *P. putida* KTQQ20, lacking three isoenzymes of FadA and FadB, as well as the 3-hydroxyacyl-ACP thioesterase PhaG, so essentially devoid of β-oxidation, was used (37) and equipped with the rhamnolipid synthesis genes *rhlAB*, resulting in strain *P. putida* KTQQ20-RL (Fig. 4).

**Fig 5:**
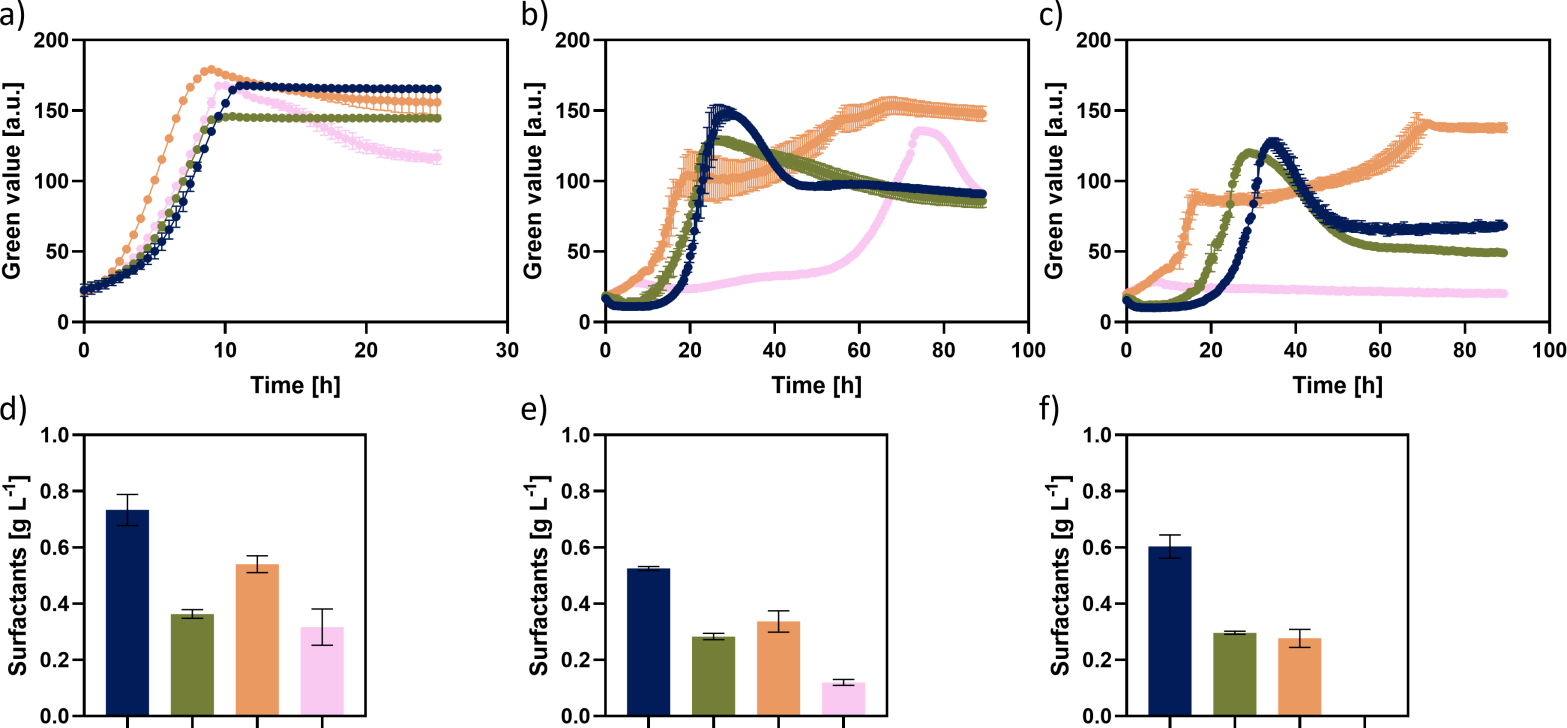
Biomass and surfactant production of β-oxidation deficient mutants on glucose and fatty acids. Growth and final surfactant concentration of *P. putida* KT2440-RL (**a and d**), *P. putida* KTQQ20-RL (**b and e**), and *P. putida* KTQQ20 ΔPP_3754-55-RL (**c and f**) on 0.33 C-mol glucose (#1, blue), 0.17 C-mol glucose (#2, green), 0.17 C-mol glucose +0.17 C-mol decanoate (#3, orange), and 0.17 C-mol decanoate (#4, pink). Cultivation was performed in 24-deep well plates. Error bars represent the standard deviation from the mean value of three biological replicates.

This strain was still able to produce biomass on decanoate (Fig. 5b), probably due to the presence of more isoenzymes taking over. M. G. Thompson et al. (49) predicted the existence of a total of six acyl-coenzyme A (CoA) ligases, seven acyl-CoA dehydrogenases, seven enoyl-CoA hydratases, four hydroxyacyl-CoA dehydrogenases, and five thiolases, which might be involved in fatty acid catabolism. Thus, a remaining copy of *fadBA*, PP_3754-55, was deleted from the genome, additionally, resulting in *P. putida* KTQQ20 ΔPP_3754-55-RL. After this additional gene deletion, growth on decanoate was abolished completely (Fig. 5c).

Rhamnolipid production of *P. putida* KTQQ20-RL and *P. putida* KTQQ20 ΔPP_3754-55-RL was investigated and compared to *P. putida* KT2440-RL to test whether rhamnolipids can be produced from glucose, from fatty acids, and from a combination of both without functional β-oxidation. While *P. putida* KT2440-RL produced rhamnolipids from all four tested substrates, *P. putida* KTQQ20-RL showed lower titers from decanoate as well as from the mixture of glucose and decanoate. *P. putida* KTQQ20 ΔPP_3754-55-RL did not produce rhamnolipids from decanoate at all, which is not surprising since no biomass was formed from decanoate as the sole carbon source. However, when grown on a mixture of glucose and decanoate, this strain exhibited a biphasic biomass formation, suggesting that both substrates could be utilized for biomass production. Nevertheless, the rhamnolipid titer reached under this condition equaled that obtained from glucose alone, indicating that the additional decanoate could not be channeled into rhamnolipid biosynthesis even when biomass formation occurred. These results suggest that rhamnolipids can only be synthesized from decanoate if β-oxidation is functional, leading to two possible explanations: Since only FadAB but not FadE is missing, trans-2-enoyl-CoA can still be formed by *P. putida* KTQQ20-RL and *P. putida* KTQQ20 ΔPP_3754-55-RL. Thus, the formation of (*R*)-hydroxyacyl-CoA via PhaJ is still possible.

The incapability of *P. putida* KTQQ20 ΔPP_3754-55-RL to produce rhamnolipids from decanoate indicates that (*R*)-hydroxyacyl-CoA is not a substrate of RhlA and probably that no trans-acylase can convert (*R*)-hydroxyacyl-CoA into (*R*)-hydroxyacyl-ACP. Fatty acids thus need to fully or at least partially undergo β-oxidation to be used for rhamnolipid biosynthesis.

**Fig 6:**
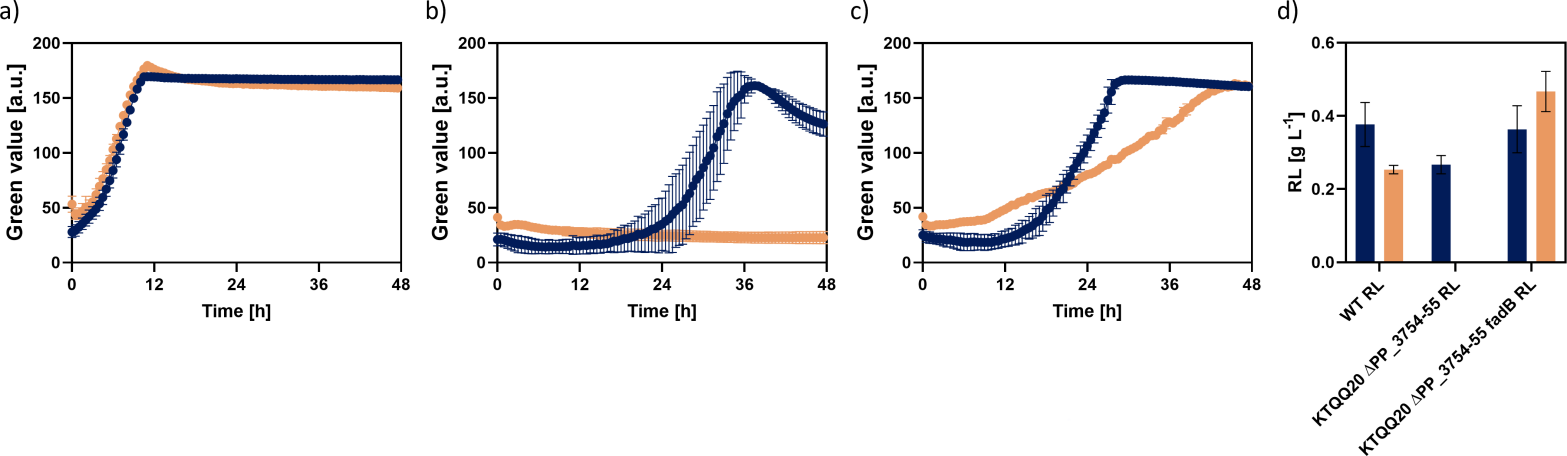
Biomass and surfactant formation of β-oxidation mutants of *P. putida* on glucose and fatty acids. Growth of *P. putida* KT2440-RL (**a**), *P. putida* KTQQ20 ΔPP_3754-44-RL (**b**), and *P. putida* KTQQ20 ΔPP_3754-44-RL-*fadB* (**c**) and the corresponding rhamnolipid titers on 0.33 C-mol glucose (blue) and 0.33 C-mol decanoate (orange) (**d**).

To investigate whether the formation of (*S*)−3-hydroxyacyl-CoA or 3-ketoacyl-CoA is sufficient for rhamnolipid biosynthesis or whether it is inevitable that fatty acids are fully degraded to acetyl-CoA, a mutant of *P. putida* KTQQ20 ΔPP_3754-55-RL was created in which the function of FadB was restored by integration of PP_2136, resulting in *P. putida* KTQQ20 ΔPP_375455RL-*fadB*. Restoring FadB by re-introducing PP_2136 also restored the ability to form biomass and rhamnolipids from fatty acids (Fig. 6). While *P. putida* KTQQ20 ΔPP_3754-55-RL was not able to produce biomass on decanoate as sole carbon source (Fig. 6b), *P. putida* KTQQ20 ΔPP_3754-55-RL-*fadB* showed biomass formation on the fatty acid, though with a very low rate of 0.04 h^−1^ (Fig. 6c). On glucose, *P. putida* KTQQ20 ΔPP_3754-55-RL-*fadB* produced rhamnolipid titers comparable to *P. putida* KT2440-RL, whereas the titers produced on decanoate were notably higher than the ones reached by the wild-type rhamnolipid producer (Fig. 6d).

The fact that restoring the activity of FadB was sufficient to re-enable rhamnolipid biosynthesis could indicate an interconnection between β-oxidation and FAS. However, since the strain is also able to produce biomass from fatty acids, this rather shows that the strain is able to fully degrade the fatty acids to acetyl-CoA, which is then used for biomass formation, indicating the activity of further FadA-like enzymes. A BLASTp analysis further revealed seven remaining gene products with similarity to FadA and eleven remaining gene products with similarity to FadB, which are still intact in *P. putida* KTQQ20 ΔPP_3754-55. These homologous enzymes are presumed to provide functional redundancy, and it is therefore likely that some of the remaining proteins with thiolase activity can compensate for the loss of FadA in this mutant strain. Still, the obtained results indicate the necessity of a functional β-oxidation to enable rhamnolipid biosynthesis from fatty acids.

To shed more light on fatty acid metabolism in *P. putida* KT2440, the transcriptome during growth on decanoate was compared to the transcriptome during growth on glucose. By this, active isoenzymes of *fadBA* could be identified (Table 4). The most active homologs and also the most upregulated genes upon growth on decanoate were PP_2047, PP_2048, and PP_2051, of which the first two are deleted in *P. putida* KTQQ20. In contrast to these results, M. G. Thompson et al. (49) found by random barcode transposon sequencing PP_2136 and PP_2137 to be the primary homologs of *fadB* and *fadA*. These two genes, which are also deleted in the mutant, only exhibited a moderate overexpression upon growth on the fatty acid here, whereas PP_2214 and PP_2215 did not exhibit a significant change in gene expression. Furthermore, PP_3754 and PP_3755, which have been deleted in this study, were shown to be very active during growth on decanoate compared to growth on glucose. However, PP_3282 and PP_3283, which also represent isoenzymes to *fadBA,* were found to be upregulated during growth on decanoate and might be an explanation for the remaining activity of FadB in *P. putida* KTQQ20 ΔPP_3754-55.

**TABLE 4:** Differentially expressed genes involved in fatty acid metabolism^*a*^

Gene name	Gene product	Locus	log2 fold change
N/A	3-Hydroxyacyl-CoA dehydrogenase family protein	PP_2047	−7.99
N/A	Acyl-CoA dehydrogenase	PP_2048	−7.78
*fadA*	3-Ketoacyl-CoA thiolase (thiolase I)	PP_2051	−6.93
N/A	Acyl-CoA dehydrogenase family protein	PP_0370	−4.81
*bktB*	Beta-ketothiolase BktB	PP_3754	−4.75
*hbd*	3-Hydroxybutyryl-CoA dehydrogenase	PP_3755	−4.54
*fadB*	Enoyl-CoA hydratase/3-hydroxyacyl-CoA dehydrogenase	PP_2136	−3.76
*pcaF-II*	Beta-ketoadipyl-CoA thiolase subunit beta	PP_2137	−3.55
*aceA*	Isocitrate lyase	PP_4116	−3.37
*fadD-I*	Long-chain-fatty-acid/CoA ligase	PP_4549	−2.93
N/A	Acyl-CoA dehydrogenase	PP_0368	−2.29
*glcB*	Malate synthase G	PP_0356	−2.18
*paaF*	Enoyl-CoA hydratase-isomerase	PP_3284	−1.86
*paaG*	2-(1,2-Epoxy-1,2-dihydrophenyl)acetyl-CoA isomerase	PP_3283	−1.29
*pcaF-I*	Beta-ketoadipyl-CoA thiolase	PP_1377	2.11
N/A	(*R*)−3-Hydroxydecanoyl-ACP:CoA transacylase	PP_1408	2.12

^*a*^ Growth of *P. putida* KT2440 on decanoate was compared to growth on glucose. N/A, not available. Full data set available in Table SA2.

These findings indicate that multiple isoenzymes of *fadBA* contribute to fatty acid metabolism in *P. putida* KT2440, with certain homologs, particularly PP_3282 and PP_3283, potentially compensating for the loss of genes in mutants like *P. putida* KTQQ20, explaining why degradation of fatty acids is still possible.

In contrast, *fadE* was not found to be differentially expressed on decanoate than on glucose in *P. putida* KT2440. Furthermore, while two of the three copies of *phaJ* (PP_4552 and PP_0580) did not show significant differential gene expression on decanoate compared to glucose, the third copy (PP_4817) showed 2.5-fold expression on decanoate compared to glucose. Thus, irrespective of the presence of decanoate, a substrate for β-oxidation, the transcription is largely maintained.

### ^13^C labeling reveals a complete breakdown of fatty acids to acetyl-CoA

**Fig 7:**
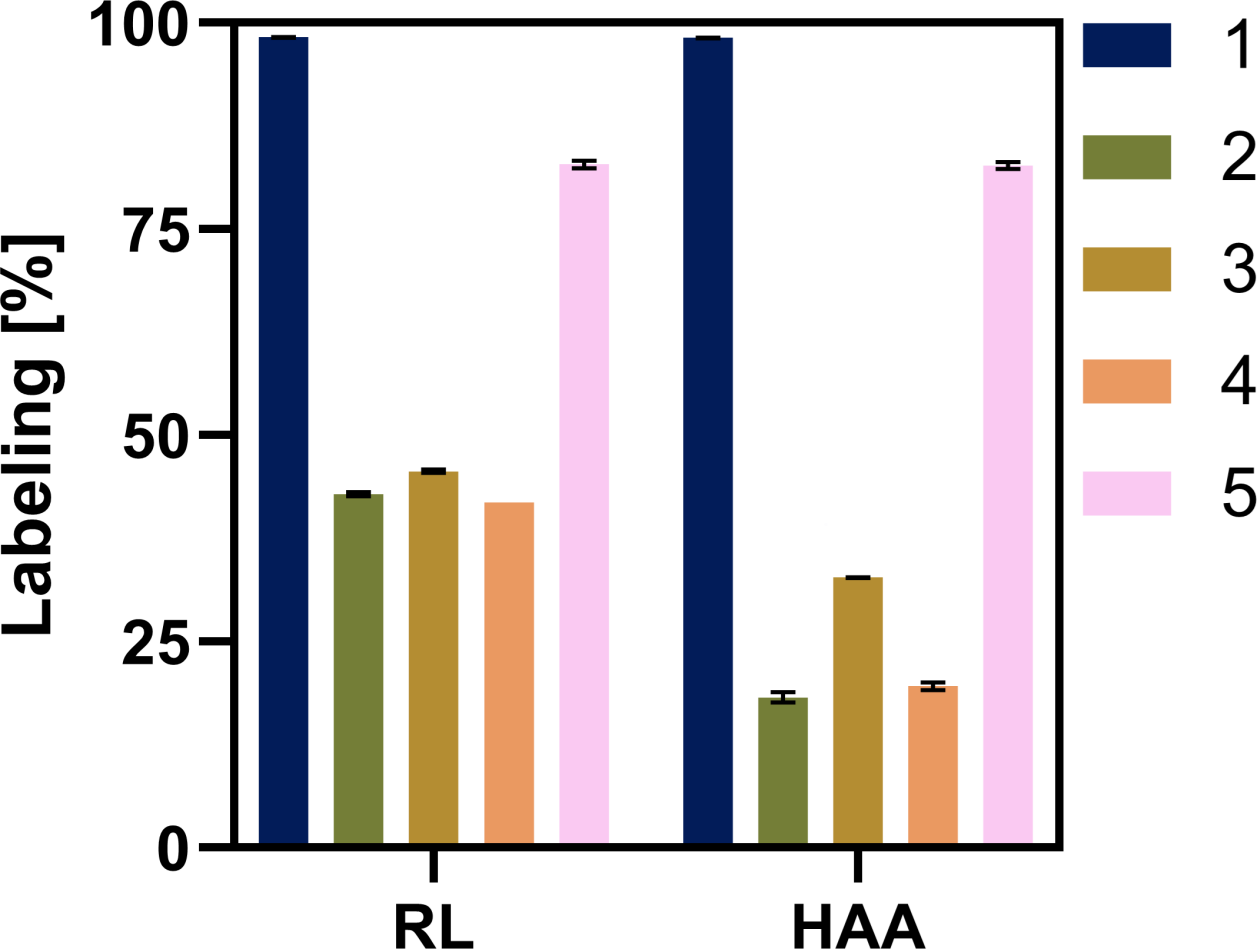
Summed FL on different carbon source(s). Summed FL of Rha-C10-C10 (RL) and C10-C10 (HAA) in samples of *P. putida* KT2440 SK4 on 0.33 C-mol ^13^C-glucose (#1, blue), 0.17 C-mol ^13^C-glucose + 0.17 C-mol octanoate (#2, green), 0.17 C-mol ^13^C-glucose + 0.17 C-mol decanoate (#3, brown), 0.17 C-mol ^13^C-glucose + 0.17 C-mol dodecanoate (#4, orange), and of *P. putida* KTQQ20 ΔPP_3754-55 on 0.17 C-mol ^13^C-glucose + 0.17 C-mol dodecanoate (#5, pink).

**Fig 8:**
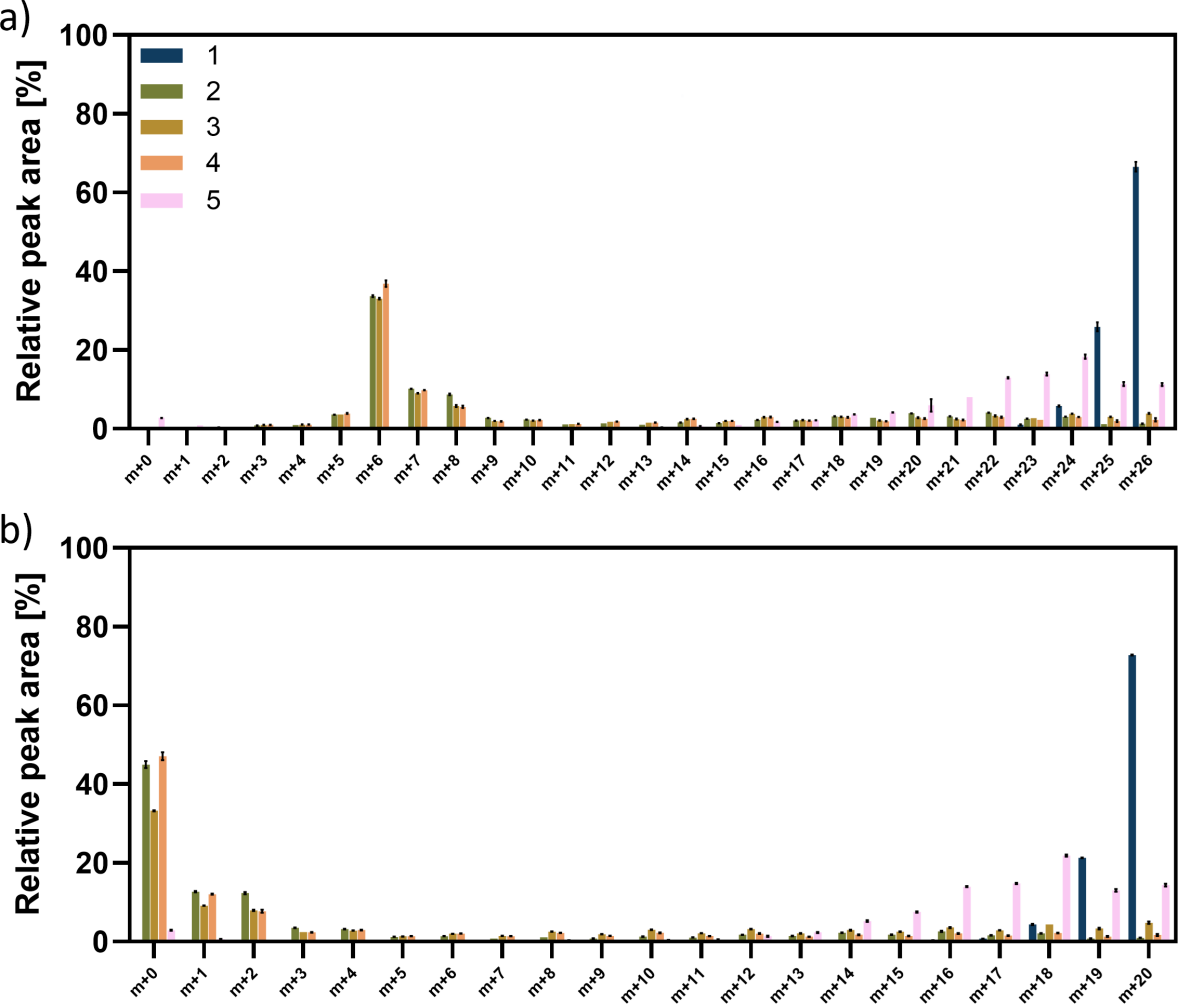
Peak areas of Rha-C10-C10 and HAA-C10-C10 in *P. putida* strains on ¹³C-glucose and fatty acids. Peak areas of masses detected for the rhamnolipid Rha-C10-C10 (**a**) and the HAA C10-C10 (**b**) in samples of *P. putida* KT2440 SK4 on 0.33 C-mol ^13^C-glucose (#1, blue), 0.17 C-mol ^13^C-glucose + 0.17 C-mol octanoate (#2, green), 0.17 C-mol ^13^C-glucose + 0.17 C-mol decanoate (#3, brown), 0.17 C-mol ^13^C-glucose + 0.17 C-mol dodecanoate (#4, orange), and of *P. putida* KTQQ20 ΔPP_3754-55 on 0.17 C-mol ^13^C-glucose + 0.17 C-mol dodecanoate (#5, pink). Error bars represent the standard deviations from the mean value of three biological replicates.

Further insights into the biosynthesis of rhamnolipids in *P. putida* KT2440 SK4 were obtained by a ^13^C-labeling experiment with a co-feed of equi-C-molar ratios of ^13^C-glucose and ^12^C-fatty acids, that is, octanoate, decanoate, and dodecanoate. The ^13^C labeling of the produced rhamnolipids was investigated using LC-MS. As a control, *P. putida* KT2440 SK4 was cultivated on 0.33 C-mol ^13^C-glucose. Since Rha-C_10_-C_10_ and HAA C_10_-C_10_ represent by far the most abundant species of mono-RLs and HAAs, data for these molecules were analyzed in detail (Fig. 8). Data for the congeners Rha-C_8_-C_10_, HAA C_8_-C_10_, Rha-C_10_-C_12_, HAA C_10_-C_12_, and Rha-C_10_-C_12:1_, HAA C_10_-C_12:1_, which show a comparable FL and a comparable distribution of peak intensities for the different analyzed masses, can be found in the supporting information (Figure A 2–A 5). Since a specific rhamnolipid or HAA congener elutes at a specific time (independent of its ^13^C content), EICs of each investigated species, including every possible ^13^C content (Table A1), were plotted using the parameters explained before. Peak areas were obtained using the Genesis peak integration algorithm. The analysis of rhamnolipids produced in the control experiment, that is, from 0.33 C-mol ^13^C-glucose, revealed that 67% of the rhamnolipids were fully labeled (m + 26), while 26% included one unlabeled C-atom (m + 25), 6% contained two unlabeled C-atoms (m + 24), and 1% contained three unlabeled C-atoms (m + 23), resulting in a total share of 98% labeled C-atoms (FL) (Fig. 7). HAAs also exhibited a FL of 98% (Fig. 7).

As expected, rhamnolipids and HAAs were synthesized from the only available substrate, ^13^C-glucose, and thus consisted almost exclusively of labeled C-atoms. The share of 2% unlabeled C-atoms can be explained by the isotopic purity of the used substrate, which is 99%, indicating that 99% of all C-atoms are labeled, and unlabeled C-atoms are contained in the inoculation biomass, which was 1% of the final biomass concentration. Thus, when a fully labeled substrate was used, the resulting rhamnolipids were fully labeled, too (which means a FL of 98%).

Rhamnolipid production of *P. putida* KT2440 SK4 on 0.17 C-mol glucose +0.17 C-mol octanoate (C_8_)/decanoate (C_10_)/dodecanoate (C_12_) resulted in a different labeling pattern but was comparable for all three fatty acids, independent of the chain length. For each substrate combination, the most abundant molecular mass found was 509, corresponding to six labeled C-atoms, which applies to 33%–37% of the molecules. Analyzing the labeling in the HAA after MS/MS fragmentation of the molecule, which cleaved the rhamnose moiety, confirmed that these six labels are located in the rhamnose molecule (Fig. 8). Since a mixture of a sugar and a fatty acid was used as a substrate, it was expected that the rhamnose moiety was synthesized from fully labeled glucose and thus, was also fully labeled, resulting in at least six labeled C-atoms in Rha-C_10_-C_10_ (m + 6). However, all masses from 508 u, that is, a C_10_-C_10_ rhamnolipid with five labeled C-atoms (m + 5), to 529 u, that is, a fully labeled C_10_-C_10_ rhamnolipid (m + 26), were detected, though with much lower intensities than the one with a mass of 509 (m + 6), corresponding to six labeled C-atoms. The peaks at *m/z* = 508 can again be explained by the 99% labeled C-atoms in the substrate and C-atoms derived from the inoculation biomass and the probably herein-contained PHA molecules: the probability for each C-atom to be labeled is then 0.99 · 0.99 = 0.98. The probability that all C-atoms in the rhamnose molecules are labeled is 0.98^6^ = 0.886. Multiplying this probability by the number of C-atoms in the molecule results in 0.886 · 6 = 5.3 labeled C-atoms in the rhamnose molecule, yielding a mass of 508 u (m + 5). *m/z* values larger than 509 resulted from more than six labeled C-atoms in Rha-C_10_-C_10_. The second and third most abundant species were found to be *m/z* = 510 (m + 7), that is, seven labeled C-atoms, corresponding to 9.0%–10.1% of the molecules, and *m/z* = 511 (m + 8), equaling eight labeled C-atoms, found in 5.6%–8.7% of the molecules. These results indicate that labeled C-atoms have also been found in the hydroxy fatty acid chains, indicating that a part of the acetyl-CoA pool was derived from the labeled glucose. More importantly for this study, the fatty acids were apparently not directly incorporated into rhamnolipid molecules as intact chains. Instead, they appear to undergo at least partial β-oxidation; that is, at least one or several cycles of β-oxidation must occur to shorten the chains, as carbon atoms derived from labeled glucose were also detected in the hydroxy fatty acid moieties. However, whether β-oxidation proceeds completely to acetyl-CoA, or whether C4 or C6 acyl-CoAs are directly incorporated into rhamnolipids cannot be determined at this stage. The majority of the acetyl-CoA pool, however, seemed to be unlabeled, leading to a summed FL of 42%–46% in rhamnolipids and 18%–33% in HAAs only (Fig. 7). Taken together, the data indicate that the rhamnose moiety was synthesized from the available glucose, resulting in higher summed FL in rhamnolipids than in HAAs, while the hydrophobic moiety was mostly synthesized from unlabeled C-atoms derived from the fatty acid substrates.

Additionally, the labeling of rhamnolipids produced by *P. putida* KTQQ20 ΔPP_3754-55-RL on 0.17 C-mol ^13^C-glucose + 0.17 C-mol dodecanoate was investigated. In total, 83% of the carbon atoms in the rhamnolipids were found to be labeled. Since the mutant lacks FadBA and, thus, was assumed to be deficient in β-oxidation, the produced rhamnolipids were expected to be labeled, and the results should be in accordance with the labeling pattern found for *P. putida* KT2440 SK4 on ^13^C-glucose only. However, the labeling was lower for the mutant, and molecules with *m/z* = 518, corresponding to nine labeled C-atoms, to *m/z* = 529, indicating fully labeled molecules, were found. 18% of the molecules were to carry 24 labeled C-atoms, representing the largest fraction, followed by 14% with 23 labeled C-atoms and 13% with 22 labeled C-atoms. The 17% unlabeled carbon atoms found in the rhamnolipids imply that carbon atoms derived from the unlabeled fatty acids were incorporated into the biosurfactants, indicating a residual activity of β-oxidation. This residual activity would also explain why restoring FadB resulted in restored biomass and rhamnolipid formation from fatty acids.

Furthermore, labeling of the amino acids serine (Ser), alanine (Ala), leucine (Leu), aspartate (Asp), and glutamate (Glu) produced during the cultivation of the plasmid-based rhamnolipid-producer *P. putida* KT2440 pPS05 in M9 medium containing ^13^C_6_-glucose and ^12^C-fatty acids was investigated using GC-MS. Amino acids are synthesized from several central carbon metabolism intermediates. Thus, amino acid labeling can provide information on whether they are built from ^13^C_6_ glucose or unlabeled fatty acids.

**Fig 9:**
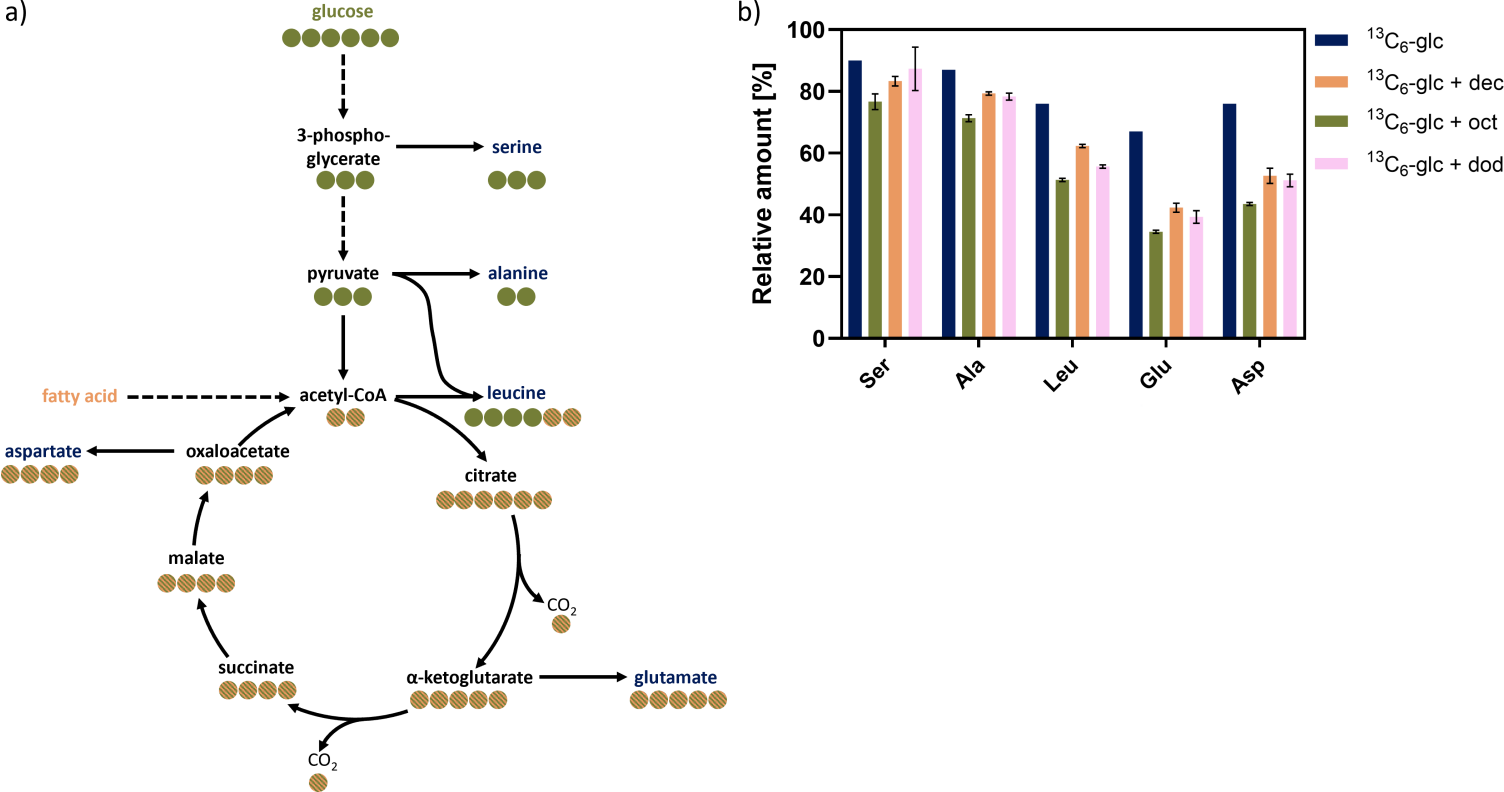
Labeling of amino acids. (**a**) Synthesis of amino acids serine, alanine, leucine, glutamate, and aspartate from glucose and fatty acids. Circles indicate the number of carbon atoms in each molecule. Filled green circles derive from glucose, green and orange hatched circles indicate carbon atoms of unknown origin. (**b**) Relative amount of ser390 + 432, ala 260, leu 344, glu 432 + 474, and asp 418 + 460 from 0.333 C-mol U-^13^C_6_ glucose (orange), 0.17 C-mol U-^13^C_6_ glucose and 0.17 C-mol ^12^C-decanoate (blue), 0.17 C-mol U-^13^C_6_ glucose and 0.17 C-mol ^12^C-octanoate (green), and 0.17 C-mol U-^13^C_6_ glucose and 0.17 C-mol ^12^C-dodecanoate (pink).

While serine is synthesized from the ED-intermediate 3-phosphoglycerate (3-PG), alanine is synthesized from pyruvate (Fig. 9). Both are expected to be built from glucose and, thus, are assumed to be fully labeled. In contrast, leucine is built from pyruvate and acetyl-CoA, the latter of which can also be obtained from fatty acids, too. Thus, leucine contains four C-atoms, which are labeled in any case since they derive from pyruvate and therefore from glucose, plus two C-atoms which are unlabeled if the acetyl-CoA derives from fatty acid degradation. A labeling of leucine of 60%, therefore, indicates that the acetyl-CoA is derived from full degradation of fatty acids. Glutamate and aspartate are synthesized from TCA intermediates: while glutamate is synthesized from α-ketoglutarate, aspartate is built from oxaloacetic acid. Thus, both can contain C-atoms derived from either glucose or fatty acids, which enter the TCA cycle via acetyl-CoA. The share of labeled C-atoms is determined by the share of acetyl-CoA derived from glucose and fatty acid catabolism.

When cultivated on glucose only, the FL was found to be the highest for serine and alanine (90%) (Fig. 9b). The labeling of leucine, glutamate, and aspartate was found to be 80%. The surprisingly low overall percentage of labeling in the samples is explained by unlabeled biomass from the pre-culture. When half of the available carbon was replaced by unlabeled decanoate, the labeling was reduced in all investigated amino acids (Fig. 9b). The highest percentage of labeling was found for serine, which was only slightly lower compared to the cultivation on ^13^C_6_-glucose only. The labeling for alanine was reduced to 80%, the labeling for leucine was reduced to 62%, the labeling of aspartate was reduced to 53%, and the lowest labeling was detected for glutamate, which only reached 42%.

FL was reduced noticeably in amino acids derived from acetyl-CoA or the TCA cycle, that is, when carbon atoms can also be derived from fatty acid degradation. While alanine is completely derived from pyruvate, leucine is derived from pyruvate and acetyl-CoA to two-thirds and one-third, respectively. If the acetyl-CoA pool consisted only of unlabeled C-atoms derived from fatty acid degradation, the expected FL of leucine would be 66% of that of alanine. Since the labeling found in alanine was 80% only, a labeling of 53%, that is, 0.8 * 0.66 * 100, was expected for leucine. In the experiments with ^13^C_6_-glucose and unlabeled decanoate, a labeling of 62% was found in leucine, which correlates to 78% of the labeling found in alanine (80%), thus indicating that 21% of unlabeled C-atoms were derived from decanoate, which has been broken down to acetyl-CoA. The share of labeled carbon in leucine was thus higher than expected (66%) if the acetyl-CoA pool consisted completely of unlabeled molecules, indicating that acetyl-CoA was partially generated from the labeled glucose, too. Glutamate was expected to show a labeling corresponding to 3/5 of the labeling of alanine if the acetyl-CoA pool consisted completely of unlabeled molecules, since two of the five C-atoms in glutamate derive from acetyl-CoA. The experimentally determined labeling amount in glutamate was slightly lower than the theoretical labeling amount. The slight difference between the theoretical and the observed labeling probably results from anaplerotic reactions using unlabeled CO_2_ from the air, for example, pyruvate carboxylase synthesizing oxaloacetate from pyruvate and CO_2_. Aspartate was expected to show half of the labeling amount of alanine, corresponding to 40% labeled C-atoms. The amount of ^13^C detected by GC-MS exceeds this theoretical value by 16%. This means that more labeled carbon atoms were incorporated, indicating that the acetyl-CoA pool is not only made up of carbon atoms derived from degradation of unlabeled fatty acids but also of labeled carbon atoms derived from U-^13^C_6_ glucose. Results obtained from cultivations on 0.17 C-mol U-^13^C_6_ glucose and 0.17 C-mol ^12^C-octanoate and 0.17 C-mol U-^13^C_6_ glucose and 0.17 C-mol ^12^C-dodecanoate resulted in comparable results with the lowest labeling detected on 0.17 C-mol U-^13^C_6_ glucose and 0.17 C-mol ^12^C-octanoate for all investigated amino acids (Fig. 9).

On the whole, the amount of labeling found in the amino acids serine, alanine, leucine, glutamate, and aspartate strongly indicates that fatty acid substrates are broken down to acetyl-CoA prior to being used for cellular metabolism, as well as rhamnolipid biosynthesis, and are not incorporated into rhamnolipids as a whole. The acetyl-CoA pool mainly consists of unlabeled carbon, derived from the fatty acid substrate, but also includes labeled molecules, derived from ^13^C-glucose, explaining the higher label in rhamnolipid/HAA molecules and amino acids than expected if acetyl-CoA were exclusively formed from unlabeled fatty acid substrates. These results thus strongly suggest that the lipid moiety of rhamnolipids is synthesized from acetyl-CoA via FAS.

## DISCUSSION

This study aimed to elucidate rhamnolipid biosynthesis and the involvement of the two central carbon metabolism cycles, β-oxidation and FAS. Analysis of the stereochemistry of rhamnolipids suggests that the acyltransferase RhlA only accepts (*R*)-configured activated hydroxy-fatty acids since rhamnolipids in (*R*)-configuration were found exclusively in samples of *P. aeruginosa* PAO1, as well as in samples of the recombinant producer *P. putida* KT2440 SK4, irrespective of the substrates. The same configuration was found in the 3-hydroxy-fatty acids of rhamnolipids and PHA, produced by *P. aeruginosa* before (14, 17, 22). While β-oxidation delivers activated hydroxy-fatty acids in (*S*)-configuration, FAS delivers activated hydroxy-fatty acids in (*R*)-configuration. However, this does not exclude β-oxidation as the main supplier of the hydrophobic rhamnolipid moiety since *Pseudomonas* features the (*R*)-specific enoyl-coenzyme A (CoA) hydratase PhaJ, converting (*S*)-3-hydroxyacyl-CoA to (*R*)−3-hydroxyacyl-CoA, and the substrate of RhlA has not yet been identified without any doubt to be CoA- or ACP-activated. Moreover, experiments with β-oxidation deficient mutants performed in this study revealed that the enzyme responsible for HAA formation, RhlA, only accepts ACP-activated hydroxy-fatty acids, and that *P. putida* KT2440 probably does not contain an enzyme with transacylase activity able to convert hydroxyacyl-CoA to hydroxyacyl-ACP. Combined with the identified stereoconfiguration, this implies that the substrate of RhlA is delivered by FAS as (*R*)-3-hydroxyacyl-ACP. Investigation of the labeling of rhamnolipids and proteinogenic amino acids synthesized from ^13^C_6_-glucose and fatty acids further indicates that fatty acids are degraded to acetyl-CoA and thus constitute the primary source for the acetyl-CoA pool, which is used for the synthesis of HAAs.

Taken together, all these results suggest that the rhamnolipid lipid moiety is formed from acetyl-CoA via fatty acid synthesis, independent ofrom the applied substrate, and that fatty acid substrates must be broken down to acetyl-CoA via β-oxidation and cannot be incorporated into rhamnolipids in any other way, at least in the recombinant producer *P. putida* KT2440 SK4. Biochemical enzyme assays, which were not performed in the context of this study, could further support the conclusions drawn here.

FAS was also found to be the supplier of the HAA precursor molecules from several substrates in the native rhamnolipid producer *B. thailandensis* E264 (21). The monomers of PHA, polyesters that have a similar precursor to that of HAAs, consist exclusively of (*R*)-hydroxycarboxylic acids in *P. putida* (50), thus also sharing the stereochemistry with HAAs. However, PHA precursors, dependent on the applied substrate, are either produced from FAS as (*R*)-hydroxyacyl-ACP or from β-oxidation as (*S*)-hydroxyacyl-CoA and channeled toward PHA synthesis by 3-hydroxyacyl-ACP thioesterase PhaG and the medium-chain length fatty acid-CoA ligase AlkK or the stereospecific trans-enoyl-CoA hydratase, PhaJ, respectively (51). Interestingly, since T. Tiso et al. (35) showed that a deletion of *phaG* even had a positive effect on rhamnolipid biosynthesis in *P. putida* KT2440, and it was found here that rhamnolipid synthesis was not possible from fatty acids in a strain deficient in FadAB, none of these pathways seems to be involved in the synthesis of HAAs. These results show that although precursors of PHA and HAA molecules share a high similarity, biosynthesis differs notably. Thus, biosynthesis pathways have to be investigated specifically for each product, and such important information cannot necessarily be transferred from a structurally related molecule to another.

Identification of the pathways involved in HAA and rhamnolipid biosynthesis in *P. putida* KT2440 paves the way for more specific metabolic engineering with the aim of generating an enhanced recombinant rhamnolipid production strain. Now, targeted interventions into the strain’s metabolism can be established to eliminate potential bottlenecks and direct the carbon flux toward HAA and rhamnolipid biosynthesis. This work thus contributes to the aim of an economic rhamnolipid biosynthesis with a non-pathogenic host.

## SUPPLEMENTAL MATERIAL

Table SA2 aem.00930-25-s0001.xlsx RNA-Seq data.

Supplemental material aem.00930-25-s0002.docx Detailed information on labeled HAAs and rhamnolipids.

ASM does not own the copyrights to Supplemental Material that may be linked to, or accessed through, an article. The authors have granted ASM a non-exclusive, world-wide license to publish the Supplemental Material files. Please contact the corresponding author directly for reuse.
