# Supplementary material for: Heterologous rhamnolipid biosynthesis by P. putida KT2440—ACP dependency and the role of fatty acid metabolism
Source: Appl Environ Microbiol. 2025 Oct 21;91(11):e00930-25. doi: 10.1128/aem.00930-25 (PMC12628675; doi:10.1128/aem.00930-25)
Supplement: Supplemental material — Detailed information on labeled HAAs and rhamnolipids. [file aem.00930-25-s0002.docx]

**Heterologous rhamnolipid biosynthesis by *P. putida* KT2440 –**

**ACP-Dependency and the role of fatty acid metabolism**

- **Supplemental Material -**


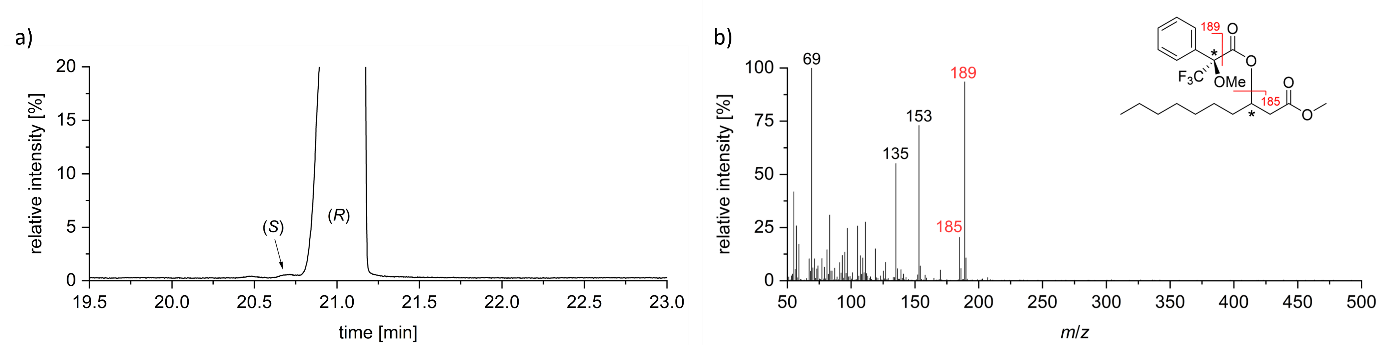


Figure A 1: Excerpt of the GC-MS chromatogram of the hydrolyzed, methylated and with MTPA derivatized rhamnolipids produced by *P. putida* grown on glucose with the (*R*)-enantiomer of 3-OH-10:0 at 21.0 min and (*S*)-enantiomer at 20.7 min (a). GC-MS mass spectrum of the MTPA derivative of the (*R*)-enantiomer of 3-OH-10:0 (t_R_ = 21.0 min) produced by *P. putida* from glucose (b). The diagnostic fragment ions (*m/z* 189 and *m/z* 185) indicate the MTPA moiety and the chain length of original 3-hydroxy fatty acid. A similar mass spectrum was obtained for the (*S*)-enantiomer (t_R_ = 20.7 min).


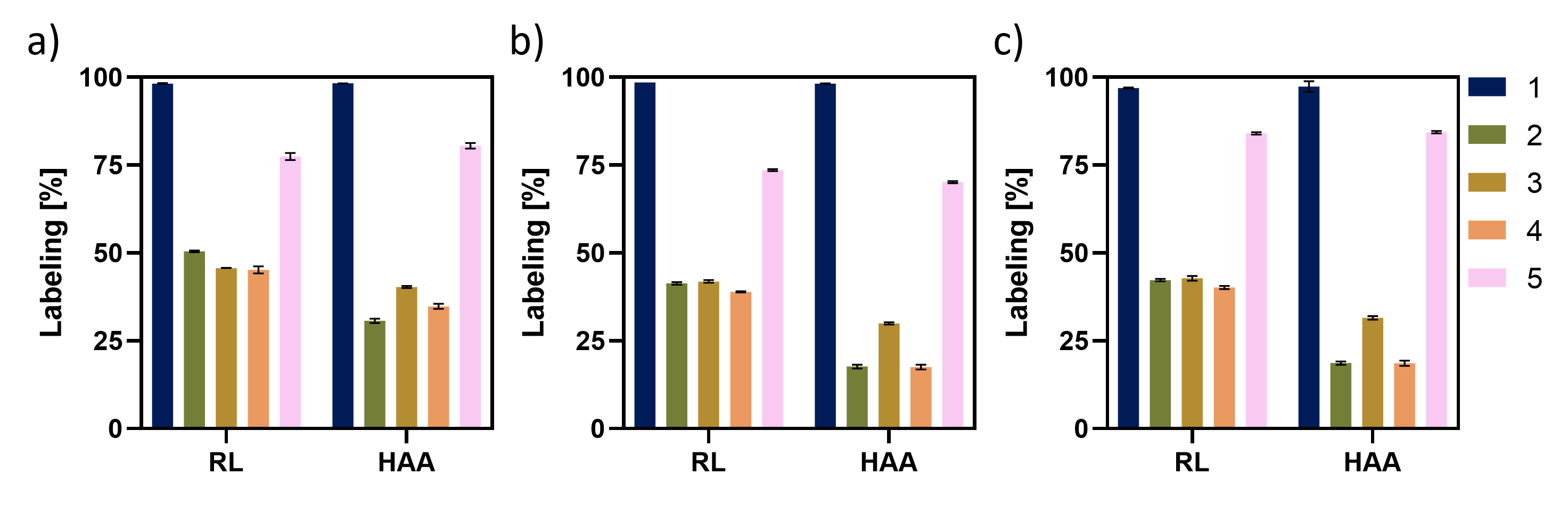


Figure A 2: Summed fractional labeling on different carbon source(s). Summed fractional labeling of Rha-C8-C10 (RL) and C8-C10 (HAA) (a), Rha-C10-C12 (RL) and C10-C12 (HAA) (b) and Rha-C10-C12:1 (RL) and C10-C12:1 (HAA) (c) in samples of *P. putida* KT2440 SK4 on 0.33 C-mol ^13^C-glucose (#1, blue), 0.17 C-mol ^13^C-glucose + 0.17 C-mol octanoate (#2, green), 0.17 C-mol ^13^C-glucose + 0.17 C-mol decanoate (#3, brown), 0.17 C-mol ^13^C-glucose + 0.17 C-mol dodecanoate (#4, orange) and of *P. putida* KTQQ20 ΔPP_3754-55 on 0.17 C-mol ^13^C-glucose + 0.17 C-mol dodecanoate (#5, pink).


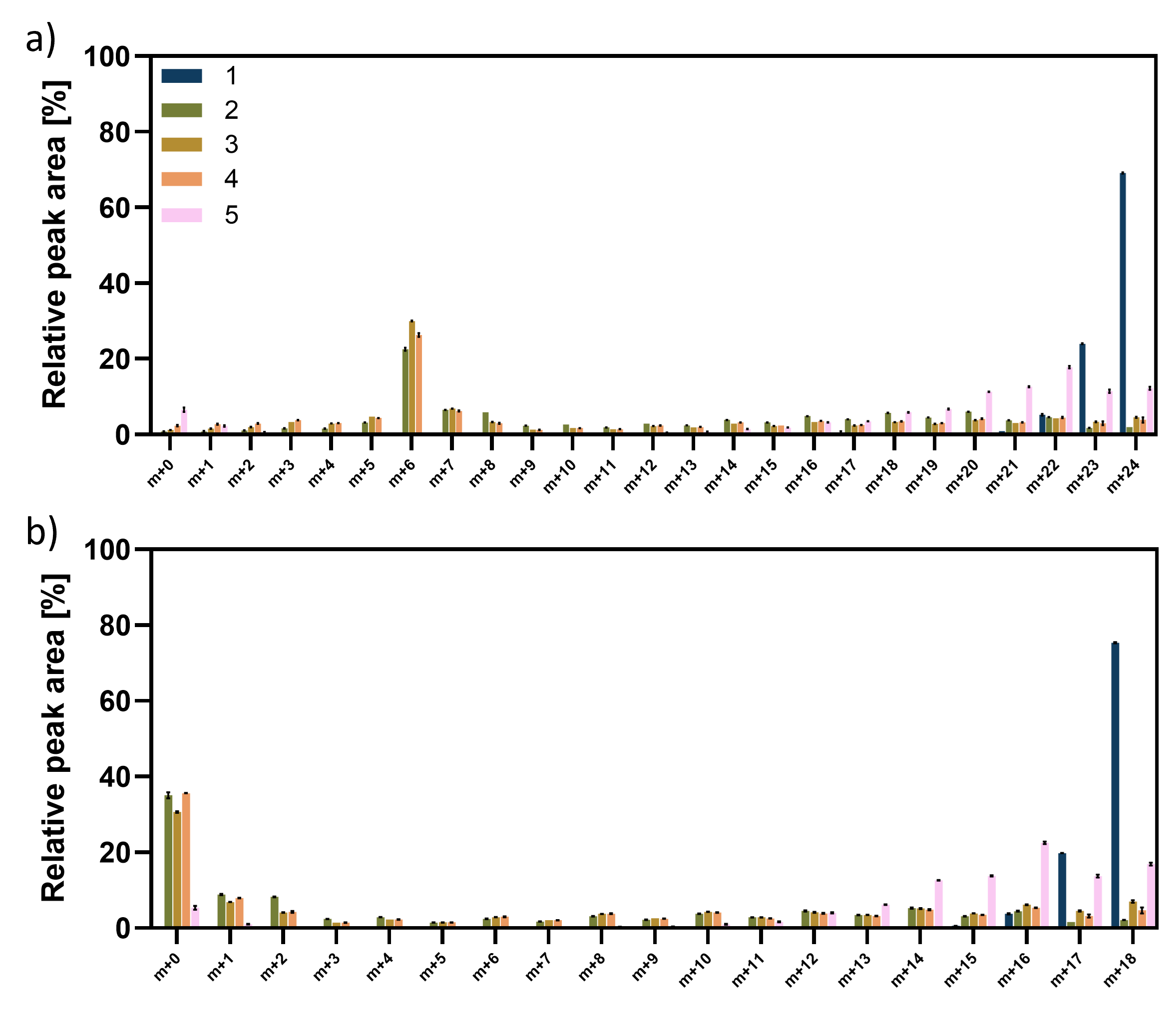


Figure A 3: Peak areas of Rha-C8-C10 and HAA-C8-C10 in *P. putida* strains on ¹³C-glucose and fatty acids. Peak area of masses detected for the rhamnolipid Rha-C8-C10 (a) and the HAA C8-C10 (b) in samples of *P. putida* KT2440 SK4 on 0.33 C-mol ^13^C-glucose (#1, blue), 0.17 C-mol ^13^C-glucose + 0.17 C-mol octanoate (#2, green), 0.17 C-mol ^13^C-glucose + 0.17 C-mol decanoate (#3, brown), 0.17 C-mol ^13^C-glucose + 0.17 C-mol dodecanoate (#4, orange) and of *P. putida* KTQQ20 ΔPP_3754-55 on 0.17 C-mol ^13^C-glucose + 0.17 C-mol dodecanoate (#5, pink). Error bars represent the standard deviations from the mean value of three biological replicates.


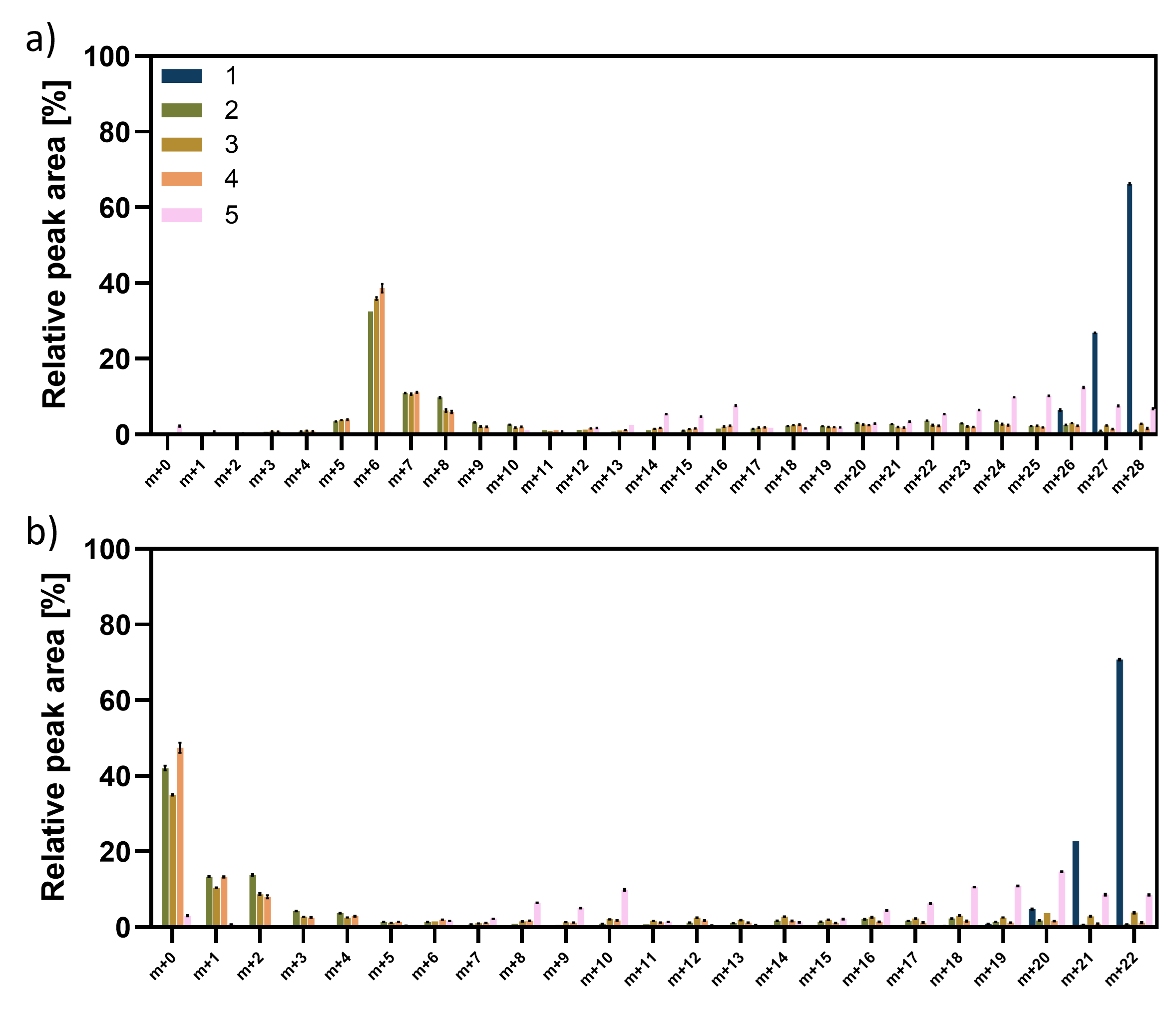


**Figure A 4: Peak areas of Rha-C10-C12 and HAA-C10-C12 in *P. putida* strains on ¹³C-glucose and fatty acids.** Peak area of masses detected for the rhamnolipid Rha-C10-C12 (a) and the HAA C10-C12 (b) in samples of *P. putida* KT2440 SK4 on 0.33 C-mol ^13^C-glucose (#1, blue), 0.17 C-mol ^13^C-glucose + 0.17 C-mol octanoate (#2, green), 0.17 C-mol ^13^C-glucose + 0.17 C-mol decanoate (#3, brown), 0.17 C-mol ^13^C-glucose + 0.17 C-mol dodecanoate (#4, orange) and of *P. putida* KTQQ20 ΔPP_3754-55 on 0.17 C-mol ^13^C-glucose + 0.17 C-mol dodecanoate (#5, pink). Error bars represent the standard deviations from the mean value of three biological replicates.


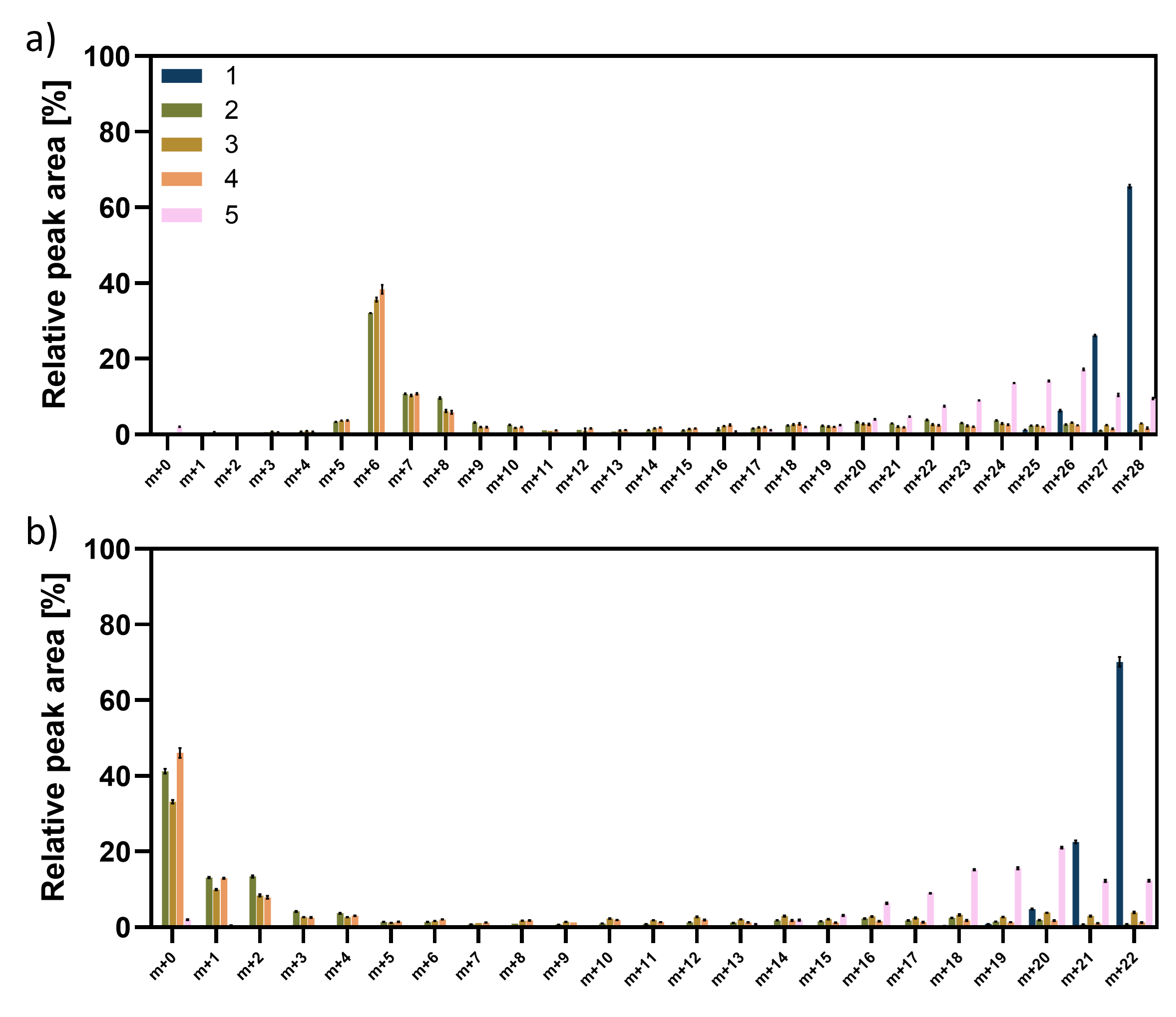


Figure A 5: Peak areas of Rha-C10-C12:1 and HAA-C10-C12:1 in *P. putida* strains on ¹³C-glucose and fatty acids. Peak area of masses detected for the rhamnolipid Rha-C10-C12:1 (a) and the HAA C10-C12:1 (b) in samples of *P. putida* KT2440 SK4 on 0.33 C-mol ^13^C-glucose (#1, blue), 0.17 C-mol ^13^C-glucose + 0.17 C-mol octanoate (#2, green), 0.17 C-mol ^13^C-glucose + 0.17 C-mol decanoate (#3, brown), 0.17 C-mol ^13^C-glucose + 0.17 C-mol dodecanoate (#4, orange) and of *P. putida* KTQQ20 ΔPP_3754-55 on 0.17 C-mol ^13^C-glucose + 0.17 C-mol dodecanoate (#5, pink). Error bars represent the standard deviations from the mean value of three biological replicates.

Table A 1: Theoretical *m*/*z* values (deprotonated molecule [M-H]^-^) of the major HAA and rhamnolipid congeners with all different labeling variants.

| HAA C_8_-C_10_ | | Rha-C8-C10 | | HAA C_10_-C_10_ | | Rha-C_10_-C_10_ | | HAA C_10_-C_12_ | | Rha-C_10_-C_12_ | | HAA C_10_-C_12:1_ | | Rha-C_10_-C_12:1_ | |
| --- | --- | --- | --- | --- | --- | --- | --- | --- | --- | --- | --- | --- | --- | --- | --- |
| labeled C-atoms | theoretical mass | labeled C-atoms | theoretical mass | labeled C-atoms | theoretical mass | labeled C-atoms | theoretical mass | labeled C-atoms | theoretical mass | labeled C-atoms | theoretical mass | labeled C-atoms | theoretical mass | labeled C-atoms | theoretical mass |
| 0 | 329.2333 | 0 | 475.2913 | 0 | 357.2646 | 0 | 503.3226 | 0 | 385.2959 | 0 | 531.3539 | 0 | 383.2803 | 0 | 529.3382 |
| 1 | 330.2367 | 1 | 476.2946 | 1 | 358.2680 | 1 | 504.3259 | 1 | 386.2993 | 1 | 532.3572 | 1 | 384.2837 | 1 | 530.3416 |
| 2 | 331.2401 | 2 | 477.2980 | 2 | 359.2714 | 2 | 505.3293 | 2 | 387.3027 | 2 | 533.3606 | 2 | 385.2870 | 2 | 531.3449 |
| 3 | 332.2434 | 3 | 478.3013 | 3 | 360.2747 | 3 | 506.3326 | 3 | 388.3060 | 3 | 534.3639 | 3 | 386.2904 | 3 | 532.3483 |
| 4 | 333.2468 | 4 | 479.3047 | 4 | 361.2781 | 4 | 507.3360 | 4 | 389.3094 | 4 | 535.3673 | 4 | 387.2937 | 4 | 533.3516 |
| 5 | 334.2501 | 5 | 480.3080 | 5 | 362.2814 | 5 | 508.3393 | 5 | 390.3127 | 5 | 536.3706 | 5 | 388.2971 | 5 | 534.3550 |
| 6 | 335.2535 | 6 | 481.3114 | 6 | 363.2848 | 6 | 509.3427 | 6 | 391.3161 | 6 | 537.3740 | 6 | 389.3004 | 6 | 535.3583 |
| 7 | 336.2568 | 7 | 482.3147 | 7 | 364.2881 | 7 | 510.3460 | 7 | 392.3194 | 7 | 538.3773 | 7 | 390.3038 | 7 | 536.3617 |
| 8 | 337.2602 | 8 | 483.3181 | 8 | 365.2915 | 8 | 511.3494 | 8 | 393.3228 | 8 | 539.3807 | 8 | 391.3071 | 8 | 537.3650 |
| 9 | 338.2635 | 9 | 484.3214 | 9 | 366.2948 | 9 | 512.3527 | 9 | 394.3261 | 9 | 540.3840 | 9 | 392.3105 | 9 | 538.3684 |
| 10 | 339.2669 | 10 | 485.3248 | 10 | 367.2982 | 10 | 513.3561 | 10 | 395.3295 | 10 | 541.3874 | 10 | 393.3138 | 10 | 539.3717 |
| 11 | 340.2702 | 11 | 486.3282 | 11 | 368.3015 | 11 | 514.3595 | 11 | 396.3328 | 11 | 542.3908 | 11 | 394.3172 | 11 | 540.3751 |
| 12 | 341.2736 | 12 | 487.3315 | 12 | 369.3049 | 12 | 515.3628 | 12 | 397.3362 | 12 | 543.3941 | 12 | 395.3205 | 12 | 541.3785 |
| 13 | 342.2769 | 13 | 488.3349 | 13 | 370.3082 | 13 | 516.3662 | 13 | 398.3395 | 13 | 544.3975 | 13 | 396.3239 | 13 | 542.3818 |
| 14 | 343.2803 | 14 | 489.3382 | 14 | 371.3116 | 14 | 517.3695 | 14 | 399.3429 | 14 | 545.4008 | 14 | 397.3273 | 14 | 543.3852 |
| 15 | 344.2837 | 15 | 490.3416 | 15 | 372.3150 | 15 | 518.3729 | 15 | 400.3463 | 15 | 546.4042 | 15 | 398.3306 | 15 | 544.3885 |
| 16 | 345.2870 | 16 | 491.3449 | 16 | 373.3183 | 16 | 519.3762 | 16 | 401.3496 | 16 | 547.4075 | 16 | 399.3340 | 16 | 545.3919 |
| 17 | 346.2904 | 17 | 492.3483 | 17 | 374.3217 | 17 | 520.3796 | 17 | 402.3530 | 17 | 548.4109 | 17 | 400.3373 | 17 | 546.3952 |
| 18 | 347.2937 | 18 | 493.3516 | 18 | 375.3250 | 18 | 521.3829 | 18 | 403.3563 | 18 | 549.4142 | 18 | 401.3407 | 18 | 547.3986 |
|  |  | 19 | 494.3550 | 19 | 376.3284 | 19 | 522.3863 | 19 | 404.3597 | 19 | 550.4176 | 19 | 402.3440 | 19 | 548.4019 |
|  |  | 20 | 495.3583 | 20 | 377.3317 | 20 | 523.3896 | 20 | 405.3630 | 20 | 551.4209 | 20 | 403.3474 | 20 | 549.4053 |
|  |  | 21 | 496.3617 |  |  | 21 | 524.3930 | 21 | 406.3664 | 21 | 552.4243 | 21 | 404.3507 | 21 | 550.4086 |
|  |  | 22 | 497.3650 |  |  | 22 | 525.3963 | 22 | 407.3697 | 22 | 553.4276 | 22 | 405.3541 | 22 | 551.4120 |
|  |  | 23 | 498.3684 |  |  | 23 | 526.3997 |  |  | 23 | 554.4310 |  |  | 23 | 552.4153 |
|  |  | 24 | 499.3718 |  |  | 24 | 527.4031 |  |  | 24 | 555.4344 |  |  | 24 | 553.4187 |
|  |  |  |  |  |  | 25 | 528.4064 |  |  | 25 | 556.4377 |  |  | 25 | 554.4221 |
|  |  |  |  |  |  | 26 | 529.4098 |  |  | 26 | 557.4411 |  |  | 26 | 555.4254 |
|  |  |  |  |  |  |  |  |  |  | 27 | 558.4444 |  |  | 27 | 556.4288 |
|  |  |  |  |  |  |  |  |  |  | 28 | 559.4478 |  |  | 28 | 557.4321 |
